# Proxy-based accelerated discovery of Fischer–Tropsch catalysts†
†Electronic supplementary information (ESI) available: Details of synthesis, analysis and testing, validation experiments for high-throughput XRD and gas treatment, details of statistical analysis and calculations, tabulation of synthesis parameters and XRD results, alternatives to Fig. 3 highlighting different data points, FTS testing results displayed graphically. See DOI: 10.1039/c4sc02116a



**DOI:** 10.1039/c4sc02116a

**Published:** 2014-10-01

**Authors:** Paul Boldrin, James R. Gallagher, Gary B. Combes, Dan I. Enache, David James, Peter R. Ellis, Gordon Kelly, John B. Claridge, Matthew J. Rosseinsky

**Affiliations:** a Department of Chemistry , University of Liverpool , L69 7ZD , UK . Email: m.j.rosseinsky@liverpool.ac.uk; b Johnson Matthey PLC , P. O. Box 1, Belasis Avenue, Billingham , Cleveland , TS23 1LB , UK; c Johnson Matthey PLC , Blount’s Court, Sonning Common , Reading , RG4 9NH , UK

## Abstract

Development of heterogeneous catalysts for complex reactions such as Fischer–Tropsch synthesis of fuels is hampered by difficult reaction conditions, slow characterisation techniques such as chemisorption and temperature-programmed reduction and the need for long term stability. High-throughput (HT) methods may help, but their use has until now focused on bespoke micro-reactors for direct measurements of activity and selectivity. These are specific to individual reactions and do not provide more fundamental information on the materials. Here we report using simpler HT characterisation techniques (XRD and TGA) along with ageing under Fischer–Tropsch reaction conditions to provide information analogous to metal surface area, degree of reduction and thousands of hours of stability testing time for hundreds of samples per month. The use of this method allowed the identification of a series of highly stable, high surface area catalysts promoted by Mg and Ru. In an advance over traditional multichannel HT reactors, the chemical and structural information we obtain on the materials allows us to identify the structural effects of the promoters and their effects on the modes of deactivation observed.

## Abstract 2

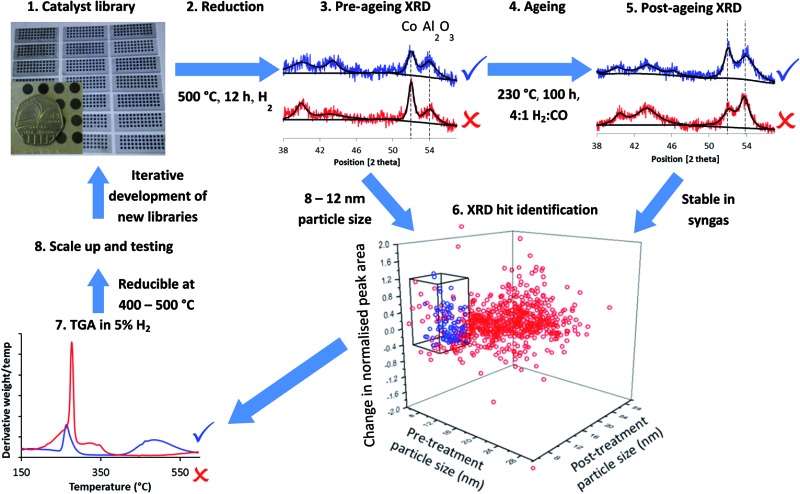
High-throughput XRD and TGA are used to screen hundreds of candidate Fischer–Tropsch synthesis catalyst samples per month for particle size, reducibility and stability under operating conditions. A series of highly stable catalysts based on Co-Ru-Mg-Al_2_O_3_ are identified.

## Introduction

Heterogeneous catalysis is a key technology in creating modern society,^1^ and improvements will be required to continue increasing worldwide living standards in the face of resource depletion, population increase and climate change. However, development of heterogeneous catalysts is slowed by deficiencies in theory, meaning that it is virtually impossible to predict whether a particular change will improve a catalyst. Experimentally, development can be hampered by the difficulties of rapidly measuring quantities such as active metal surface area, particle size and reducibility, practical difficulties measuring rates and selectivities towards different products and the need for long testing times to assess stability.

Fischer–Tropsch synthesis (FTS), which creates hydrocarbons from a mixture of carbon monoxide and hydrogen (syngas), is a particularly difficult reaction to develop catalysts for, due to the complex nature of the reaction, the mixture of chemically similar products, and the extremely long testing times required for an accurate measure of activity and stability.^2–4^

A possible solution to these problems is high-throughput (HT) experimentation, involving the rapid synthesis and characterisation of materials coupled with a screening test to assess the suitability of the materials.^5–7^ However, in catalysis, this has focused on increasing testing throughput,^8–12^ without exploiting traditional techniques such as temperature programmed reduction (TPR) and chemisorption which provide information on catalyst reducibility,^13,14^ active metal particle size^15,16^ and metal surface area^17,18^ that have been shown to be linked to high activity and selectivity over a century of catalysis research and are therefore able to be used as proxies. Additionally, multichannel reactors provide little or no chemical or structural information on the catalysts themselves meaning that extensive off-line characterisation is required to understand the behaviour of the materials and provide a foundation for informed improvement of the catalysts.

Industrially, for reactions such as FTS it is often of more importance to improve the lifetime of the catalysts rather than the activity. FTS plants are typically run at intermediate per pass conversions in order to protect the catalyst and reduce localised heating caused by the highly exothermic nature of FTS.^19^ The activity typically declines by 1% per week,^20^ and to compensate for this the temperature of the reactor is increased, which usually has the effect of reducing the selectivity towards the desired heavier hydrocarbons and increasing the production of methane.^21^ Shell report that a shutdown to regenerate their catalyst is required every 9–12 months.^22^ Desired total catalyst lifetimes have been given as four years by BP^23^ or five years by Shell.^24^ Due to the expense and scarcity of FTS catalyst components such as Co and Ru, as well as the cost of plant shutdowns it is desirable to increase this lifetime.

There are several deactivation routes for cobalt FTS catalysts – sintering,^25–27^ irreversible oxidation and formation of metal–support compounds,^28,29^ carbon deposition^26,30,31^ and poisoning.^32^ The main bottlenecks in investigation of FTS catalysts are measurement of Co surface area *via* chemisorption (throughput of one sample per day per machine) and testing, which requires 60–100 h to provide a stable baseline and further tests of several months to estimate the long term stability of the catalyst.

Herein, we report the use of HT XRD and TGA to gather information on particle size, surface area and reducibility both before and after an accelerated ageing test, which can be used as proxies for high activity, selectivity and stability *in lieu* of full, time-consuming FTS testing. Advances in automated XRD analysis have allowed synchrotron radiation to be used for real-time process monitoring for catalysts,^33,34^ but to our knowledge this is the first report of a lab X-ray source for HT serial screening of metallic nanoparticle catalysts. Meanwhile, HT-TGA has been used for screening catalyst activity at low temperatures,^35^ but this is the first report of its use for HT-TPR. These techniques together give an increase in throughput of catalyst characterisation of up to 100 times, providing data on thousands of hours of stability testing time per month. Importantly, in contrast to high-throughput microreactor testing, structural and chemical information is obtained on all materials, allowing us to identify not only whether a catalyst is active or stable, but also how and why. This rapidly identifies materials meeting proxy stability and activity criteria and reduces valuable testing time wasted on intrinsically unsuitable materials.

## Results and discussion

**Fig. 1:**
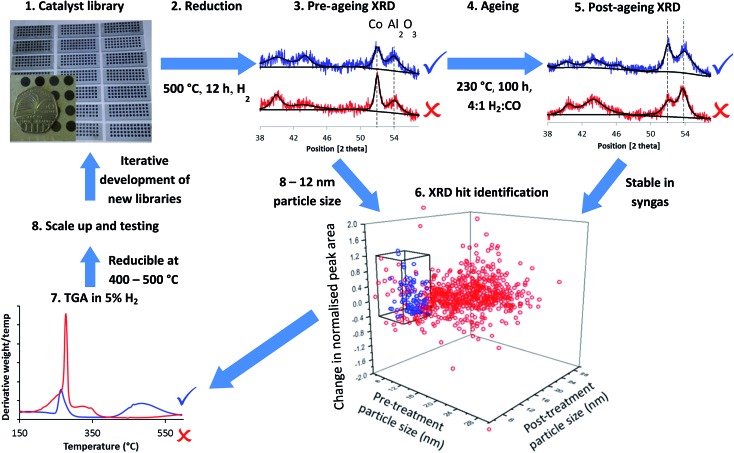
Accelerated discovery of stable Fischer–Tropsch catalysts. Catalyst libraries are synthesised by impregnation, producing oxides (step 1) and reduced to form the Co metal particles in well plates (step 2) before XRD is performed (step 3) to measure the Co metal particle size and amount of Co metal with respect to the support. Libraries are aged in syngas (step 4) before having XRD performed again (step 5). Sintering and loss of metallic Co is measured by comparison of XRD before and after the ageing treatment which acts as a proxy for stability. Hits identified (step 6) are in blue and are samples with Co particles in the range 8–12 nm before and after ageing treatment and which are stable with respect to loss of metallic Co (as measured by the difference in normalised peak area before and after treatment). XRD patterns in red are misses due to too large particle size (step 3) and too great a loss in metallic Co (step 5). Hits from XRD have their reducibility assessed by TGA in 5% H_2_–N_2_ (step 7). The blue trace is reducible at 400–500 °C while the red trace will be poorly reduced at 500 °C. Samples are selected for scale-up and catalytic testing (step 8) followed by feedback to inform the design of the next library.

Fig. 1 summarises the workflow used, which we developed for 144 distinct samples per month including a repeat for each sample. In step 1, we synthesised libraries *via* a largely automated incipient wetness procedure. We chose to focus on incipient wetness because it maps well on to a large number of industrial FTS catalyst preparation protocols, although our protocol measures stability and activity through proxies and so it could be applied to FTS catalysts emerging from any synthesis protocol, not simply the one we chose. The samples were chosen for these libraries based on hypothesis testing and exploring new compositional and processing space, as discussed below. This synthesis methodology allowed us to produce the 144 samples in one day with around 6 person-hours of work. We reduced the samples in ceramic 48 well plates (step 2) in a furnace allowing concurrent reduction of 3 plates, followed by XRD analysis to determine particle size from the peak broadening in step 3. A number of methods have been used to determine particle size in FTS catalysts, for example TEM,^36–39^ XPS,^37,38^ hydrogen chemisorption^37–39^ and XRD.^36,39^ Each method makes different assumptions, but the results deliver consistent particle sizes.^37–39^ Cobalt FTS catalysts show a normal inverse relationship between particle size and activity above 8 nm. Below this they begin to show a decrease in turnover frequency, while a maximum in C_5+_ selectivity is seen around 8 nm, although this varies depending on the support.^36–39^ In addition, experiments^40^ and modelling^41^ have shown that smaller particles are likely to be unstable under FTS conditions. Because of this we also assume that cobalt too highly dispersed or present in too small particles to be observed by XRD is unlikely to be stable or active. For these reasons, we chose a particle size range of 8–12 nm as a proxy for high activity and selectivity.

In step 4, we aged the samples under hydrogen-rich syngas for 100 hours at 230 °C. This should increase deactivation rates compared to standard conditions for initial activity testing. In step 5 we analysed the samples again by XRD, enabling assessment in step 6 of two measures of stability under ageing conditions – change in particle size and change in the amount of metallic Co by comparing pre- and post-treatment peak widths and areas (calculated relative to support peak area). Using these two metrics we are able to assess the deactivation caused by sintering through the increase in particle size, and that caused by irreversible oxidation of cobalt and formation of cobalt carbides, both of which result in a decrease in the amount of metallic cobalt. It is important to note that this workflow can identify stable catalysts directly (from changes in the structure of the material) while activity and selectivity are only determined by proxy from a desirable particle size.

In step 7, we studied a smaller number of samples using rapid TGA in 5% H_2_–N_2_ to obtain “high-throughput TPR” (HT-TPR) traces and degrees of reduction from oxide to metal which act as a further proxy for high activity.

Although we ran the workflow with 144 samples per month, if a dedicated diffractometer and larger or more furnaces were available this would increase to 850 samples per month. For step 7, HT-TPR, a fully dedicated machine could run 1200 samples per month. This approach would be ideal as a screening tool before a multichannel reactor capable of testing dozens of samples per month.

We validated the XRD (steps 3 and 5), reduction (step 2) and syngas treatment (step 4) steps of the workflow, by testing with well plates filled with identical samples. We report details in full in the ESI text S2, S3, Fig. S3–S5 and Table S1.† The standard deviation of particle sizes was 1.5 nm, while the standard deviation of the peak area ratio was 0.12 – throughout this work we regard these standard deviation values as being indications of the likely statistical error.

Initially we used the workflow as a series of general screens for a range of composition variables including Co loading and promoters which have received attention in the literature. These were chosen based on the Fischer–Tropsch literature, which has been reviewed recently.^42,43^ In one round of the workflow, Co loading (three levels), base metal (Fe,^44^ Mn,^45^ Mo,^46^ Mg^47^), base metal loading (four levels), and precious metal (Ru,^48^ Re^45^ or neither) were investigated using γ-Al_2_O_3_ as a support (144 samples), while another screen focused on supports with the variables of Co loading (five levels), support (γ-Al_2_O_3_, TiO_2_, SiO_2_, CeO_2_, zeolite Y), precious metal (Re, Ru or neither) (72 samples). Further similar screens involved other promoters (Ca,^49^ Ba,^50^ Zr,^51^ Zn^52^ on TiO_2_, Ni,^53^ Cu^54^ on γ-Al_2_O_3_) in combination with either Re or Ru or neither.

The baseline for comparison was a 20% Co, 0.1% Ru on γ-Al_2_O_3_ catalyst which should have good activity but deactivate relatively rapidly.^41^ This sample had an initial particle size of 13.4 nm which does not rule out having good activity (falling within one standard deviation of the upper bound proxy criterion), but this particle size increased by 2.7 nm on ageing in syngas, while the metal peak area decreased by 0.35, indicating that the stability, which is directly measured by the proxy screen, was indeed poor.

From these results we could easily rule out many combinations which would likely produce inactive catalysts, for example by the observation of very large particle sizes or the absence of Co peaks either before or after the ageing test, which would suggest respectively low surface area, poor reducibility or high instability. Importantly, in a distinct advantage over traditional HT multichannel tests, the structural and chemical information obtained allows us to know how and why certain materials were successful or unsuccessful. For example, we could ascertain that the presence of Ru or Re is important for improving reducibility and reducing particle size. We could then, in subsequent rounds of the workflow, further examine combinations which fell in or close to the desired particle size range as well as showing stability and reducibility using finer scale variation of the variables, using the chemical knowledge obtained to mitigate against failings in the initial samples and thus enhance catalyst performance. The plot in Fig. 1 shows 864 samples from an eight month period, equivalent to 86 400 hours of stability testing time in a serial approach. This rate of sample throughput is 75% of our theoretical design value, demonstrating that our HT workflow is sustainable over long periods of time.

In the initial screens, in samples using Mg as a promoter on Al_2_O_3_ the peak area of the cobalt indicated that the samples were likely to be poorly reduced, and therefore inactive unless Ru was also used to improve the reducibility. However, the samples gave particle sizes close to the target range, and were particularly stable. Thus the system Co–Ru–Mg–γ-Al_2_O_3_ was chosen for further investigation, with the aim of reducing the particle size and improving the reducibility in order to produce materials more likely to possess good activity.

As described in the methods sections, we used three variables – loading of Mg, loading of Ru, and the order of addition of the different elements. We chose values for Mg loading ranging from trace values (0.1%) to a value corresponding to close to monolayer coverage of the support (6%). For Ru loading we chose the industry standard value (0.1%), as well as values around this, to investigate if it would be feasible to reduce the loading (Ru being a major cost of an FTS plant) or if there were major benefits to increasing this. As mentioned above, a zero loading of Ru had been ruled out by our initial screening studies. Order of addition was investigated as it is underexplored in the literature in comparative studies. We selected three orders of addition: OoA 1, where the Mg is added before the Co and Ru, with a calcination at 550 °C; OoA 2, where the Mg is added first but without the calcination; and OoA 3 where the Mg is added after the Co and Ru. This produced 72 samples (*i.e.* half a month of samples, see ESI Table S2†) which we subjected to the workflow shown in Fig. 1, including XRD at steps 3 and 5 (data and calculated values can be found in ESI Tables S3 and S4, details of the analysis are given in ESI text S2†).

We analysed results from step 3 of the workflow – pre-ageing XRD – using the general linear model analysis of variance (ANOVA), which allows us to separate and quantify the contributions of the three different variables studied (and interactions between variables) to the observed initial particle size. A fuller explanation of the statistical method is given in ESI text S4.†

**Fig. 2:**
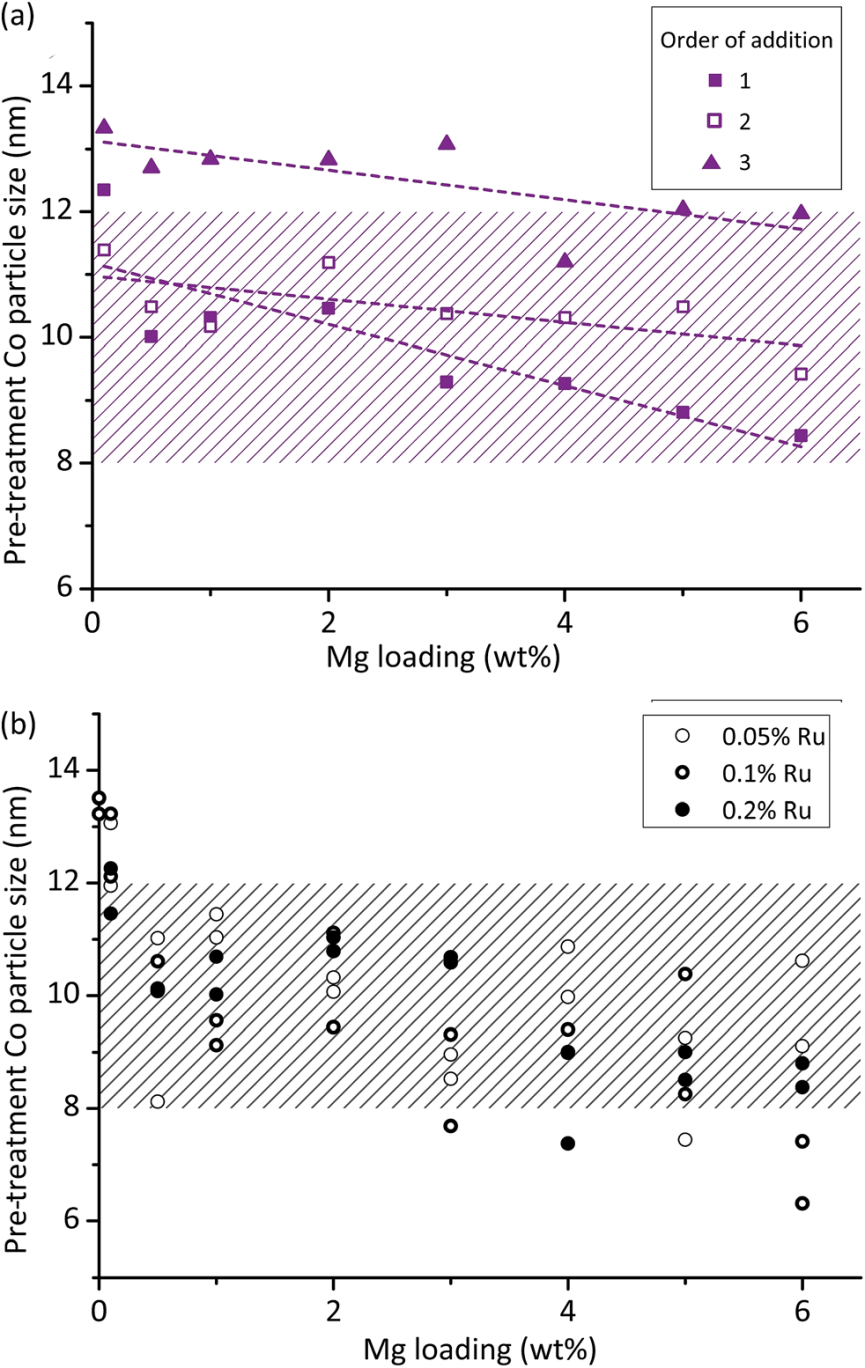
Control of cobalt metal particle sizes by catalyst synthesis protocol. The raw data was subjected to multiple linear regression which allows quantification of the effect of different synthesis variables: (a) shows the results of this analysis for the particle size data, showing that increasing Mg loading decreases particle size, while adding Mg before cobalt (OoA 2) reduces particle size, and calcining the support after addition of Mg reduces it further (OoA 1). The interaction between these two variables can also be seen, meaning that Mg loading has a larger effect on particle size when it is added before (OoA 1 and 2) rather than after (OoA 3) Co. (b) Shows particle sizes as calculated by the Scherrer equation *vs.* Mg loading for samples where Mg was added to the support and calcined before Co addition (OoA 1) showing that Ru loading has little effect on particle size. Hashed areas show 8–12 nm target particle size range.

This analysis shows that Mg loading and order of addition have the largest effects on the particle size, with the interaction between these two also being of interest. Fig. 2a shows the results, identifying that particle size decreases with increasing Mg loading, and that OoA 1 and 2 samples have a smaller particle size than OoA 3 samples. It identifies the interaction between variables, which is that increasing Mg loading decreases the particle size when Mg is added before Co in the synthesis process (OoA 2), and even more when Mg is added before Co with a high temperature calcination before Co addition (OoA 1), but not when Mg is added after Co (OoA 3). This could be due to the Mg modifying the surface of the support, which can only occur when the Mg is laid down before the Co is added. Ru loading does not have a significant effect on particle size, and this can be seen in Fig. 2b, showing how the particle size changes with Mg loading for OoA 1 samples.

As per the workflow shown in Fig. 1 we returned the well plates to the tube furnace for step 4 – ageing, followed by step 5 – measurement of XRD peak widths and areas. These steps provide information on stability over 100 hours for 144 catalysts per month – equivalent to 600 days of serial testing time. We found that order of addition, Mg loading and their interaction were again the dominant features in controlling stability. Ru loading did not affect stability.

**Fig. 3:**
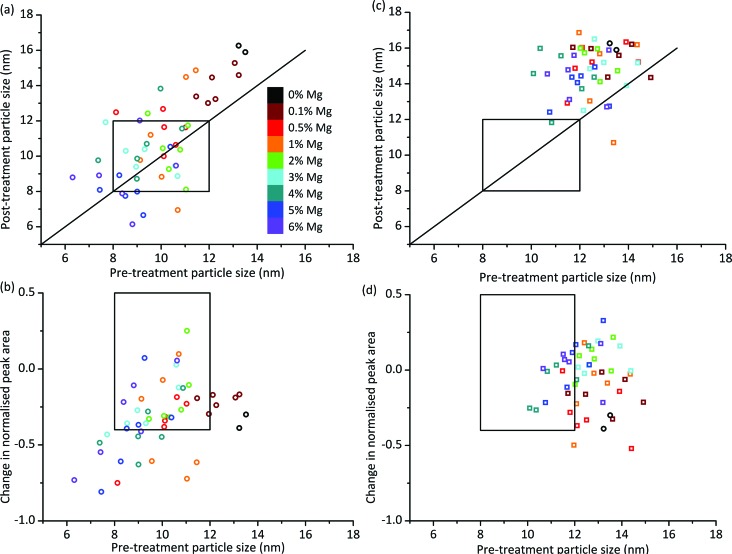
Particle size and peak area ratio changes on ageing treatment. (a) Particle sizes before and after ageing treatment for Mg added before Co and calcined (OoA 1), showing that smaller Co particles made by this route are not generally more prone to sintering than larger ones. Each Mg loading has 6 data points as there are three Ru loadings and two points per unique sample. Ru loadings are not distinguished in this figure as the regression analysis showed little effect of Ru loading. Versions with Ru loadings distinguished are shown in Fig. S9.† If points lie above the superimposed *y* = *x* line, sintering has occurred. Points below the line are likely due to error (the validation work showed a standard deviation in particle size of 1.5 nm), but could also be due to metal particle reaction with support. The box shows the hit region defined by particle sizes of 8–12 nm pre- and post-ageing treatment. (b) Change in normalised Co peak area against pre-treatment particle size for OoA 1 samples, showing that smaller particles generally lose more Co peak area than larger ones. The box shows the hit region defined by pre-treatment particle size of 8–12 nm and a change in normalised Co peak area of 0.5 to –0.4. (c) Particle sizes before and after ageing treatment for OoA 3 showing a large degree of sintering of around 3 nm. (d) Change in normalised Co peak area against pre-treatment particle size for OoA 3 samples, showing that these samples generally lose less Co peak area than OoA 1 samples, and the protective effect of Mg in this respect.

We can look at the particle size stability of the samples in more detail by plotting pre-treatment particle size against post-treatment particle size. Fig. 3a shows this plot for OoA 1 samples. Most of the points lie above the *y* = *x* line which indicates that most of the samples see an increase in particle size. On average the increase is 0.7 nm, which compares favourably with the 0% Mg samples, which increased by 2.7 nm. Some of the samples with smaller pre-treatment particle sizes lie below the *y* = *x* line, which would indicate that they have become smaller upon treatment. A possible explanation for this is that the particles have reacted with the support, therefore becoming smaller. In order to judge whether this is the case, we can compare pre- and post-treatment cobalt peak areas. Fig. 3b shows pre-treatment particle size against loss of cobalt peak area, which confirms that the samples with the smallest Co particles see the largest decreases in Co peak area. This is in broad agreement with the literature which shows that small particles are more likely to oxidise or form irreducible metal–support compounds than larger particles.^27,41^ Hence the main form of deactivation for OoA 1 samples is loss of metallic Co rather than particle growth. This deactivation mechanism is controlled by Mg loading to afford several “hits” within this family. We can see that the 0% Mg samples decrease in normalised peak area by a similar amount to samples containing Mg with much smaller particle sizes (*e.g.* 2 or 3% Mg samples), indicating that Mg has a significant protective effect.

The behaviour of OoA 3 samples was markedly different to that of the OoA 1 samples. Fig. 3c shows pre- against post-treatment particle size, indicating that the samples increase in particle size fairly uniformly, with an average increase of 3.2 nm, similar to the 0% Mg samples. Fig. 3d shows that they are relatively stable towards reaction with the support, and increasing Mg loading appears to have a stabilising effect.

We observed OoA 2 materials as intermediate between OoA 1 and OoA 3 samples, showing intermediate particle growth (on average 0.9 nm), relatively large decreases in Co peak area similar to OoA 1 samples and with a slight protective effect of higher Mg loadings similar to OoA 3. Information on these samples is shown in ESI Fig. S8 and S9.†

Combining all this information, we can begin to assess how the Mg is affecting the catalysts. The location of Mg appears to be a controlling factor in the stability of the catalysts – in OoA 1 samples it is likely that the Mg will be contained in and on the surface of the support, possibly hindering the movement of Co on the surface and reducing the amount of sintering, while in OoA 3 samples the Mg is most likely contained in and on the surface of the Co particles, possibly reducing the reactivity of the Co with the support, but not able to reduce the particle size or sintering. OoA 2 samples display a combination of these effects. The presence of Mg on the support before the addition of Co also has an important effect on reducing the particle size, and the fact that a high temperature calcination increases this effect indicates that this may be caused by “MgAl_2_O_4_”-type species.

Again we can see the benefits of the HT workflow, as we now have information on the effect of a range of composition and process variables on particle size and stability towards changes in structure in response to environmental factors. We can conclude that adding Mg after Co (OoA 3) is a poor method for producing a catalyst, giving larger particles more prone to growth. Adding Mg before Co (OoA 1 and 2) produces smaller particles, more stable towards sintering, with a calcination between adding Mg and Co (OoA 1) providing an extra reduction in particle size and further resistance to particle growth. This protective effect is likely due to some modification of the surface of the support.

Step 6 of the workflow as described in Fig. 1 involves assessment of all the XRD data to determine which samples meet our criteria for particle size as a proxy for selectivity and activity alongside changes in particle size and Co peak area measuring stability under reaction conditions. We assessed the samples based on three criteria from the two XRD steps in the workflow shown in Fig. 1 – from step 3, a pre-treatment particle size in the range 8–12 nm (initial particle size, shown in Fig. 2), from step 5 a post-treatment particle size in the range 8–12 nm (particle size growth, shown in Fig. 3a and c) and from combining the data from steps 3 and 5, a decrease in normalised Co peak area of less than 0.4 (loss of metallic Co, shown in Fig. 3b and d). Using these criteria, there were nine samples where both points fell within this region – five OoA 1 samples and four OoA 2 samples. The areas of the sample space where most points were located in the “hit” region were low-to-mid Mg loading OoA 1 samples and mid-to-high Mg loading OoA 2 samples.

**Fig. 4:**
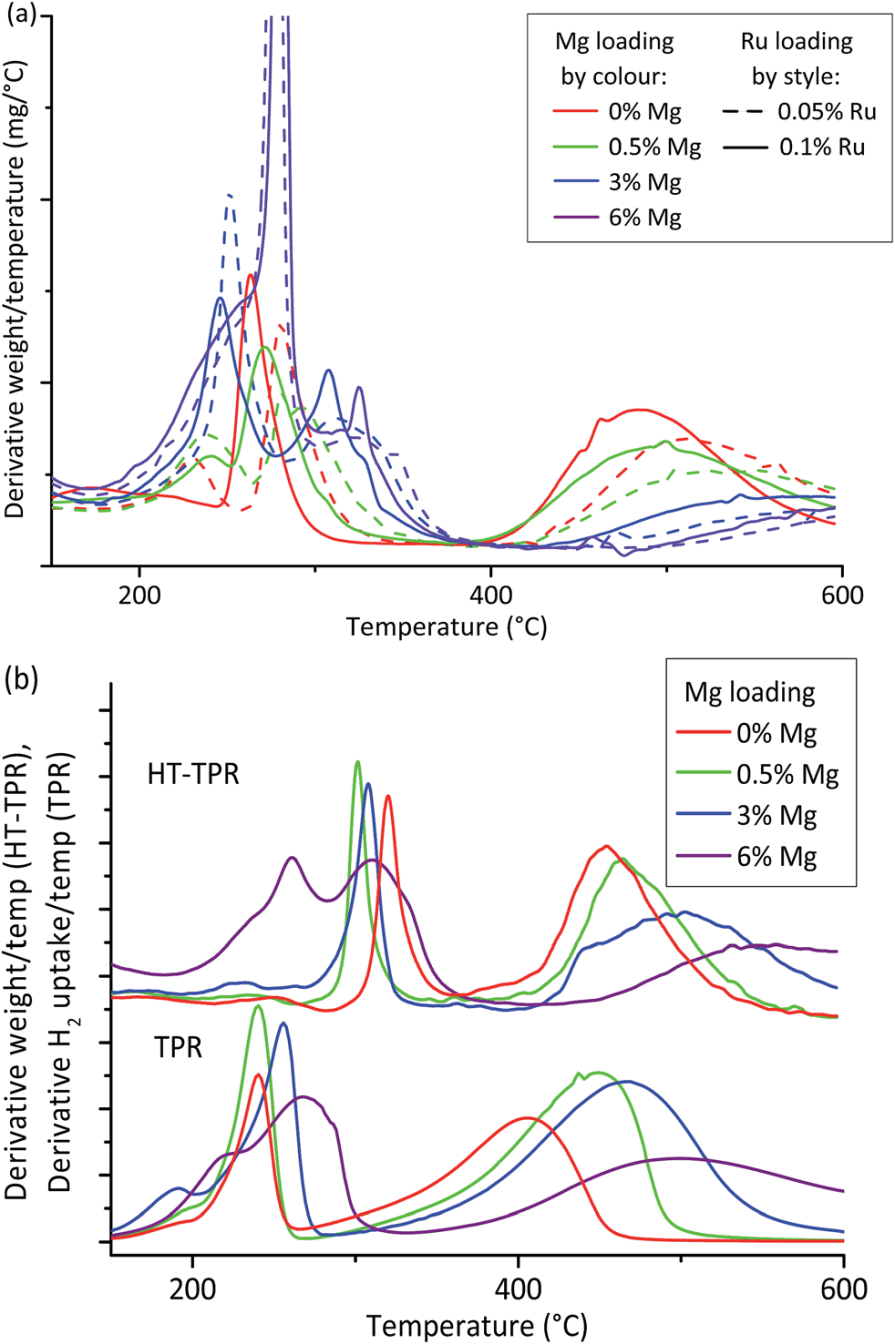
High-throughput TPR measurement of reducibility. (a) High-throughput TGA in 5% H_2_ acts as a high-throughput TPR (HT-TPR) screen to assess reducibility for OoA 1 samples. Mg lowers reducibility while Ru increases it. (b) Comparison of HT-TPR and conventional TPR traces for the scaled up samples showing that both methods show the same features, but these are shifted around 75 °C to higher temperatures in the case of the HT-TPR traces.

Since all the OoA 1 samples were in the 8–12 nm hit region and were stable towards particle growth, we chose to focus on these for characterisation by HT-TPR (step 7 in the workflow described in Fig. 1). We also investigated the effect of Ru loading. Our proxies to this point have shown no benefit in using higher loadings of the expensive Ru, but Ru is known to enhance the reduction of Co, so we wanted to assess the effect of Ru loading in more detail. HT-TPR consists of TGA in 5% H_2_–N_2_ using a dynamic heating rate and an autosampler, meaning that around 40 samples per day can be studied compared to three on a standard TPR. The results are shown in Fig. 4a.

The HT-TPR traces all had three main features. We assigned the lowest temperature peak to residual nitrate decomposition,^55^ the next peak to Co(iii)–Co(ii) reduction and the final, highest temperature, peak to Co(ii)–Co(0).^56^

Mg loading had a large effect on the behaviour of the sample, with the higher temperature reduction peak shifting to higher temperatures with increasing Mg loading. We can also see that higher loadings of Ru increase the reducibility. As a complement to the XRD “hit” criteria we can use HT-TPR as a guide to reducibility, showing that samples containing 6% Mg are likely to be very poorly reduced at the reduction conditions used in testing, while samples containing 0.1% Ru are likely to perform better than those containing 0.05% Ru.

We then chose to scale up two samples which passed all three XRD criteria, and two which failed in different ways. From the OoA 1 samples, we chose 0.5% Mg and 3% Mg as successful samples which met all three criteria, while 6% Mg failed on decrease in Co peak area, and was also shown by the HT-TPR to be poorly reduced. We also chose an OoA 3 sample containing 4% Mg which failed on particle growth but succeeded on the others. We selected a Ru loading of 0.1% since the HT-TPR results showed that Ru loading did have an important effect on reducibility. Fig. S10† highlights these samples on the particle size and stability plots. We also scaled up the baseline, 20% Co, 0.1% Ru on γ-Al_2_O_3_ (henceforth 0% Mg) which, as discussed, should have good activity but showed poor stability on our HT tests to act as a control. We carried out conventional TPR and chemisorption on these samples and tested them for FTS activity in a microreactor. Comparing the HT-TPR results with the conventional TPR results from the scaled up samples (Fig. 4b), the traces are shifted to higher temperature by around 80–100 °C in the HT-TPR, but still show all the main features and importantly, relative shifts in peaks are retained, meaning that we are able to see changes in reduction behaviour caused by changing experimental parameters with the HT-TPR technique. The key features identified in the HT workflow are thus transferred to the scaled-up samples.

**Fig. 5:**
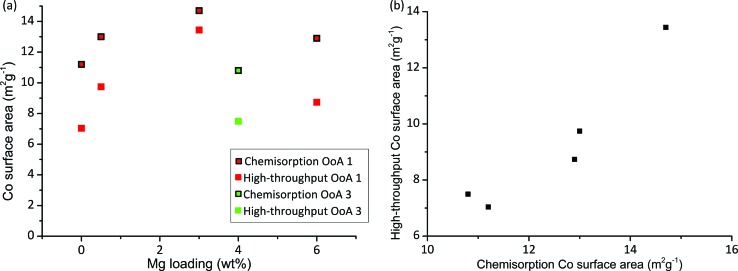
Cobalt surface area measurement by conventional and high-throughput methods. (a) Plot showing Co surface area measured by chemisorption directly and calculated high-throughput surface area, both of which show the same trends for the catalysts studied. High-throughput surface area is calculated from Co particle size measured by XRD (as shown in Fig. 2) and reducibility measured by high-throughput TGA in 5% H_2_ for samples with different Mg loadings using OoA 1 (red) and OoA 3 (green). (b) Shows the same data plotted as high-throughput surface area against surface area measured by chemisorption, showing the linear correlation.

Chemisorption with H_2_ provides a measurement of the metal surface area and for many reactions the metal surface area is directly proportional to activity. A particle size can be calculated from the surface area and degree of reduction,^57^ and therefore we can calculate a HT surface area from our XRD and HT-TPR results (details of calculation given in S5†). Fig. 5 shows HT surface area and chemisorption-based Co surface area against Mg loading. We can see that the HT surface area correlates very well with chemisorption measurements, hence Co surface area can be rapidly measured on large arrays. There is an offset between the HT surface area and the chemisorption surface area, which is likely due to the different assumptions made in the two methods (*e.g.* stoichiometry of gas adsorption, particle shape and many others), and the different reduction temperatures used. Importantly, we have correctly identified the highest metal surface area catalyst containing 3% Mg.

A conventional metal surface area measurement takes at least one full day while using a fully dedicated XRD and TGA as described in this paper could produce 1200 surface area measurements per month if the XRD was used solely to measure pre-treatment particle sizes or 850 samples per month including testing for stability.

The HT screen indicated that OoA 1 samples with low loadings of Mg should be stable under FTS conditions, and our catalytic testing results (Table 1 and ESI Fig. S11–S13†) show that low loadings of Mg do indeed impart a remarkable degree of stability to the catalysts. We tested the samples at 210, 230 and 240 °C, before returning to 210 °C. The 0% Mg catalyst, after being subjected to testing conditions at 230 °C and 240 °C, has a conversion stability of 68% compared to the 0.5% Mg and 3% Mg catalysts which have values of 84% and 99% respectively. The 4% OoA 3 sample, produced by adding the Mg after the Co rather than before as with the other samples, was selected for testing to be representative of this process for catalyst preparation. This sample failed the particle size stability test in the same way as the 0% Mg control does and thus shows a conversion stability of only 75% (Table 1). This demonstrates the effectiveness of the HT-XRD stability screen.

**Table 1:** Catalytic performance data for scaled up samples^*a*^

Mg loading (wt%)	Order of addition (OoA)	Initial interval (hours)	Final interval (hours)	Space velocity (L g^–1^ h^–1^)	Initial syngas conversion (%)	Syngas conversion stability (%)	CH_4_ selectivity stability (%)	C_5+_ selectivity stability (%)
0	N/A	99–118	237–271	13.36	26.2 ± 0.4	68 ± 2	90 ± 5	102 ± 1
0.5	1	103–120	237–271	13.37	17.4 ± 0.5	84 ± 3	108 ± 9	98 ± 3
3	1	80–118	241–271	13.38	11.9 ± 0.4	99 ± 6	89 ± 12	106 ± 6
6	1	110–121	237–271	13.48	0.0 ± 0.2	N/A	N/A	N/A
4	3	20–43	218–240	13.27	2.7 ± 0.2	75 ± 17	52 ± 17	112 ± 14

^*a*^From the high-throughput screen we successfully identified samples containing 0.5% and 3% Mg would show the highest conversion stabilities as they showed the least change in the ageing test (step 5). Further, the initial particle size tests (step 3) and reducibility (step 7) successfully indicated that these samples would show good activity. Samples containing 4% Mg and 6% Mg, which failed the proxies for activity (particle size and reducibility, steps 3 and 7 respectively) show very low activity. Initial conversion is the average during the initial period at 210 °C. Stability values for conversion and selectivity are calculated by averaging over the initial period at 210 °C and the final period at 210 °C and taking a percentage. Selectivity data is not shown for 6% Mg at 210 °C as the conversion was too low to achieve reliable values. Errors shown are standard deviations (± *σ*) of the experimental values used to calculate the averages.

As discussed earlier, our XRD screen directly measures stability, but can only be a proxy for activity and selectivity, and this can be seen by the fact that our control catalyst in fact has the highest initial activity, with addition of Mg reducing the activity. However, the catalysts we tested in our desired particle size range possessed good activity and good selectivity, and as described in the introduction, a highly stable catalyst can be more desirable than a highly active one. While it is known that highly active catalysts tend to be less stable due to localised production of heat and steam,^27^ our tests are run at low conversion and with diluted catalyst, meaning that the increased activity of the control catalyst cannot account for the decrease in stability. Since our workflow gives us information about the chemistry of the materials, we are able to hypothesise routes to improve the catalyst. In the case of the Mg catalysts, we can see from our results that a likely route for improvement would be to improve the reducibility. Indeed, the testing results indicate that the activity shows a better correlation to the temperature of reduction, as measured by the peak maximum in the TPR (Fig. S14†). This also suggests a possible route to the development of more sophisticated proxy-based screens.

## Conclusions

These results show that starting from a broad screen of around 900 samples selected according to literature understanding shown in the plot in Fig. 1, we have managed by synthesis of a more focused library of 72 subsequent catalysts to successfully identify two catalysts which, from our proxies based on initial (step 3 of the workflow) and final (step 5) particle size, stability of Co peak area (step 6) and reducibility (step 7), were correctly predicted to be both active and stable. Samples which failed the proxy criteria proved to be poor catalysts in full scale testing. These catalysts form the basis of a patent, demonstrating the ability of this workflow to identify patentable compositions in a crowded patent space.^58^ To obtain these direct catalytic testing results for all 72 samples studied in the final HT stage, which took us two weeks, would require six months of continuous testing, while for the approximately 900 samples, shown in Fig. 1, which took us eight months, would require six years. To additionally obtain cobalt surface areas and degrees of reduction would require several extra years, which would still be required even if parallel reactors were used to increase the testing throughput. Measurement of fundamental physical properties of the catalysts not only permits us to identify high performance materials, but also reveals how the materials change structurally and chemically, giving insights into factors controlling catalyst performance and thus improving our ability to further develop the materials and inform future screens. This HT proxy-based approach should be easy to generalize. We have developed the synthesis section of this workflow for incipient wetness impregnation, but it should apply to other common synthetic techniques. HT-XRD is of most use where parameters such as particle size and growth can be used as proxies for efficacy or stability as in heterogeneous catalysis, but can more generally be applied to screening materials for use in harsh conditions.
