# Supplementary material for: Proxy-based accelerated discovery of Fischer–Tropsch catalysts
Source: Chem Sci. 2014 Oct 1;6(2):935–44. doi: 10.1039/c4sc02116a (PMC5811142; doi:10.1039/c4sc02116a)
Supplement: Supplementary file 1 [file SC-006-C4SC02116A-s001.pdf]

Supplementary information

S1 – Synthesis of materials

High-throughput samples were prepared by aqueous incipient wetness impregnation using $Co(NO_3)_2.6H_2O$, (98%, Alfa Aesar), $Mg(NO_3)_2.6H_2O$ (≥ 99%, Fluka) and $Ru(NO)(NO_3)_3$ (min 31.3 % Ru, Alfa Aesar) as precursors and Puralox TH 100/150 γ-Al_2O_3 (BET surface area 145 m^2g^{-1}, pore volume 0.94 cm^3g^{-1}) as the support. 0.2 g of alumina was weighed into glass vials using a Chemspeed Accelerator SLT synthesis platform. Solutions (2.04 M $Mg(NO_3)_2$, 2.79 M $Co(NO_3)_2$, 0.0235 M $Ru(NO)(NO_3)_3$) were then dispensed into those vials using an Eppendorf epMotion 5075PC, and the volume was made up to the incipient wetness volume with distilled water. The volumes used are given in table S2. Samples were stirred manually.

72 HT samples were synthesised with 20 wt% Co, while three Ru loadings (0.05 wt%, 0.1 wt%, 0.2 wt%) and eight Mg loadings (0.1 wt%, 0.5 wt%, 1 wt%, 2 wt%, 3 wt%, 4 wt%, 5 wt% and 6 wt%) were used. Three different orders of adding the components were tested. Order of addition 1 (OoA 1) involved adding the Mg first, calcining at 550 °C for 5 h, then adding the Co and then finally the Ru. OoA 2 was identical, except the material was heated at 120 °C after Mg addition rather than 550 °C. In OoA 3, Co was added first, then Ru, and finally Mg, with only drying at 120 °C in between. Samples were dried at 120 °C for 4 h in static air in between impregnations and all materials were dried at 120 °C for 4 h followed by calcination at 250 °C for 8 h in static air once all additions were complete. All steps of the synthesis were carried out in glass vials except for calcinations at 550 °C which were performed in porcelain crucibles. A full list of samples and incipient wetness volumes used for synthesis can be found in Supplementary Table S2.

Scaled-up synthesis was carried out in the same way as the HT samples without the use of robotic platforms, with the exceptions that the Co and Ru were co-impregnated and that the mixing steps were carried out in plastic bags, while the drying and calcining steps were carried out on stainless steel trays.

Calcined samples were transferred to pyrophyllite well plates for reduction. Each plate of half the standard size defined by the Society for Biomolecular Sciences (SBS) contained 48 wells and each sample was placed in two randomly selected wells to improve accuracy. Samples in well plates were reduced under pure flowing H_2 for 12 h at 500 °C and then passivated at room temperature in 1% O_2/Ar for 2 h, both at atmospheric pressure. Before ageing treatment, samples were reduced as before and then treated for 100 h at 230 °C in a gas flow of H_2:CO ratio of 4:1 at atmospheric pressure before being passivated again. Three well plates could be reduced in the furnace at one time using a stainless steel holder (see Supplementary Figure S1).

S2 – High-throughput analysis

High-throughput XRD was performed on a Panalytical X-pert Pro diffractometer with a XYZ stage using Co Kα1/α2 (ratio = 2) radiation. The time and angle range of the XRD analysis was optimised based on a combination of being able to change the two plates in the diffractometer roughly once per day, scanning a range encompassing at least one peak each of Co metal, Co_3O_4, CoO and a support peak, and obtaining data of high enough quality to reliably fit automatically. For γ-Al_2O_3, this resolved to scans lasting 13 minutes per sample and encompassing a 2θ range of 38 – 57°. The Panalytical Operator Interface software was used to control the diffractometer for data collection, and the raw data files were named using bar codes scanned into the software before collection.

The raw data was fitted automatically using X'Pert HighScore Plus using the User Batch feature of the software. The fitting routine used the standard subroutines in HighScore Plus. First, the Search Peaks subroutine was used to find peaks in the data, followed by several Profile Fit subroutines to fit the peaks, each fitting a region of the XRD pattern containing one peak to a pseudo-Voigt peak shape. Finally, the Rich Text Report subroutine was used to output the 2θ value, FWHM, intensity and area for each peak. Each fitted XRD pattern was manually checked for obvious errors in fitting. Depending on

the dataset, typically around 95% of the XRD patterns were fitted correctly. Common errors were peaks not being detected or the profile fit algorithm diverging and returning an abnormally large FWHM value (>2.5°). This was more common in samples with a particle size less than 6 nm or with very weak peaks. In cases where the fitting routine incorrectly fitted a pattern it was manually fitted. An Excel macro was used to combine and arrange the output files from HighScore Plus into an Excel spreadsheet for further analysis.

The particle size of the Co metal phase was calculated from the line broadening of the most intense FCC Co peak using the Scherrer equation. Although there are other contributions to XRD peak broadening, empirically fitted peak width gives a good correlation to surface area[1]. Scans were taken after reduction and after ageing treatment. The amount of Co metal present was calculated by normalising the area of the Co peak against the intensity of the γ-Al_2O_3 peak at $2\theta = 53°$. The intensity rather than the area of the peak was used due to the difficulty of fitting the Al_2O_3 peak.

TGA scans (HT-TPR) were carried out on a TA Q5000IR using 5% H_2/N_2 as the sample gas flowing at 25 mL min^{-1}. Samples (approximately 5 mg) were ramped to 600 °C, holding at 600 °C for 5 min. The dynamic high resolution mode was used with a ramp rate of 50 °C min^{-1}, sensitivity of 1.0 and a resolution of 4.0.

Details of the procedures for TPR, Co surface area measurements and FTS testing are given in S6 of the SI.

S3 - XRD and gas treatment validation

S3.1 – Experimental

Well plate positions

Throughout the description of the validation samples are described in a numerical system as shown in Supplementary figures S1 and S2. Each plate has 48 samples, numbered from 1 to 12 in the first column through to 37 to 48 in the fourth column. When describing the diffraction experiments, two plates are placed in the diffractometer, with the first being samples 1 to 48, and the second being 49 to 96. The second plate is rotated 180° relative to the first, so that sample 96 on the second plate corresponds to sample 1 on the first. Columns are along the long axis of the well plate (i.e. samples 1 to 12 are column 1, 13 to 24 column 2 etc.). Rows are along the short axis (i.e. row 1 is samples 1, 13, 25, 37 etc.).

High-throughput XRD validation

To validate the acquisition of XRD measurements from across the well plates two different oxides: TiO_2 (42 m^{2}g^{-1},73 % anatase, Johnson Matthey) and Al_2O_3 (Puralox TH 100/150) - with different particle size were placed in each well of two plates and the XRD pattern acquired. XRD patterns were measured using the experimental setup, optics and counting time described in the methods section and S2 except for the 2θ range which was 40°- 60°.

Gas-treatment validation

A 20 % Co/ 0.1 % Ru/ Al_2O_3 catalyst was synthesised as described in the methods section. The material was placed in each well of three plates and put through the workflow described in paper in order to assess the effect of position in the furnace on the particle size and normalised cobalt peak area. Each plate and therefore each unique well was measured in the normal orientation and the rotated orientation and therefore each unique well was measured twice. Hence a total of 288 XRD patterns were measured. Gas treatment and XRD conditions were as described in the methods section.

S3.2 - Results

High-throughput XRD validation

The position of one peak from each material is shown in Supplementary figure S3 for each well and indicates a small systematic error associated with well position most likely to result from small height changes across the XYZ stage. Errors in the measurement of the peak position of this magnitude have only a very small effect on the final value for particle size. Importantly, there is no such measurable systematic trend in the FWHM of each peak (Supplementary figure S4). Hence the systematic error in peak position has negligible effect on the measurement of particle size. The mean FWHM for TiO_2 was 0.49° with a standard deviation of 0.04° while the FWHM for Al_2O_3 was 1.50° with a standard deviation of 0.05°. Therefore as expected the rutile peak gives a larger particle size of 21.6 nm compared to the Al_2O_3 peak which gives a particle size of 6.9 nm. The similar standard deviation of the FWHM of the two peaks indicates that the error in fitting is similar across this range of particle size. However, since the particle size depends on the reciprocal of the FWHM, the standard deviation of the *particle size* calculated from the much narrower TiO_2 peak is much larger than that of the Al_2O_3, being 1.6 nm compared to 0.2 nm for Al_2O_3. Since FWHM has negligible systematic error, the differences between the measurements for each well can be considered to arise due to random error.

Gas-treatment validation

Pre- and post- ageing treatment samples were analysed to extract peak position, FWHM, height and area of the cobalt metal peak and the Al_2O_3 support peak. Analysis of all 288 XRD patterns revealed that the mean cobalt particle size measured pre-ageing treatment was 12.4 nm and the standard deviation was 1.4 nm. After ageing treatment the particle size had increased to 13.3 nm with a standard deviation of 1.6 nm. The normalised cobalt peak area decreased from 1.02 to 0.52 after ageing treatment and the standard deviation was 0.12 in both cases. Hence an indicative error of ± 1.5 nm for particle size and ± 0.12 for normalised peak area should be considered on single measurements.

Since each plate has 48 wells and there are three positions for the well plates in the gas treatment furnace, during each reduction/ageing treatment there are 144 unique sample positions. The mean particle size and normalised cobalt peak area were calculated for each of the three plates by averaging the values from each plate in both the normal and rotated orientation therefore 96 values were averaged for each plate. The mean particle sizes for each plate (Supplementary Table S1) were close with a difference of only 0.1 nm between plates pre-ageing treatment and a maximum difference of 0.5 nm between the top and middle plate after ageing treatment indicating that the position of the plates within the furnace affects neither the initial particle size nor the sintering to much extent. The normalised cobalt peak area is similar for the three plates before ageing treatment, increasing by 0.05 on going from top to bottom. However, after ageing treatment, on going from bottom plate to the top plate, the normalised Co peak area decreases from 0.59 to 0.55 to 0.43 indicating that the plate at the top of the furnace lost more metallic cobalt than the plates lower down possibly due to heat or vapour concentration gradients. However, the difference between top and bottom is small compared to the loss of cobalt compared to the average loss caused by syngas treatment.

The change in measured properties on going downstream from row 1 to row 12 was analysed by averaging the values across all plates and columns in both normal and rotated orientation therefore 24 values were averaged for each row (as shown in Supplementary figure S5). There was no systematic effect on particle size on moving along the plate either before or after ageing treatment. Additionally there was no systematic change in normalised cobalt peak area. However, after exposure to syngas, there was a systematic decrease in normalised cobalt peak area along the well positions from 0.60 at row 1 to 0.40 at row 12. This decrease indicates that the wells further downstream lost more metallic cobalt more than those further

upstream. Since water is a product of FTS, on moving downstream the samples closer to the outlet are likely to be in a more water vapour rich atmosphere than those closest to the inlet due to the greater amount of catalyst in the region before the well in question. Water can have a profound effect on the deactivation of FTS catalysts, particularly in promoting cobalt oxidation or metal-support compound formation. Again though, this difference is small compared to the loss of cobalt caused by the syngas treatment. Lateral differences were assessed by averaging across all plates and rows therefore 72 values were averaged for each column. There was little difference in the particle sizes (≤ 0.5 nm) and normalised cobalt peak area (≤ 0.05) for the different columns of the plates, before or after ageing treatment indicating that there was little lateral inhomogeneity.

S4 - Statistical analysis

Pre- ageing treatment cobalt particle sizes and normalised cobalt peak areas extracted from XRD measurements were analysed using the general linear model to perform analysis of variance (ANOVA) to determine which factors were statistically significant. The general linear model[2] is a statistical procedure whereby a dependent variable (or variables) is regressed against multiple independent variables, with the purpose of being able to predict the dependent variable(s). The model fits the data to the equation for k independent variables $Y = \alpha + E + \beta_1X_1 + \beta_2X_2 + \ldots\beta_kX_k$, where Y is the dependent variable, X is the value of the independent variable (or transformation of the variable e.g. the square of the value), β is the weight or coefficient, E is the error and α is the intercept. A model is then produced of the form $Y = \alpha + \beta_1X_1 + \beta_2X_2 + \ldots\beta_kX_k$. For each independent variable the model outputs a P value, which quantifies the likelihood that the independent variable has no effect on the dependent variable (i.e. the value of β is zero) and calculates an ESD, an R^2 and an adjusted R^2 for the overall model. The ESD is the square root of the mean square error. An adjusted R^2 attempts to correct for the fact that increasing the number of terms in the model generally increases the R^2.

In each case, the model was reduced by sequential removal of factors and interactions with the largest P values in order to maximise the adjusted R^2 and minimise the ESD. Supplementary tables S5 – S8 show the results for the final model for pre- and post- ageing particle size and pre- and post- ageing cobalt peak area ratio. Residuals were found to be approximately normally distributed as shown in Supplementary figures S6 and S7. Figure 2a in the main text shows the results of the model plotted graphically.

S5 - Calculation of high-throughput surface area

Normally, a particle size can be calculated from the surface area and degree of reduction (from TPR) using the formula $d = aLR/S$ where d is the particle size, L is the weight loading of metal, R is the degree of reduction, S is the surface area and a is a simplification of a number of terms including density, factors relating to particle geometry which as described previously[3], can be taken to be 0.0674. We can rearrange this equation to enable us to calculate a high-throughput metal surface area using XRD particle size (from step 3 of the workflow) and degree of reduction (from step 7). Figure 5 in the main text shows high-throughput surface area and chemisorption based Co surface area against Mg loading. Our high-throughput degree of reduction represents the fraction of the total number of moles of oxygen lost compared to the theoretical amount, which was calculated from the weight loss between 150 and 500 °C. The particle size used was simply the average of the two Scherrer equation particle sizes for each sample from the high throughput screen.

S6 – Full scale catalyst characterisation and testing

Temperature-programmed reduction was carried out in an Altamira AMI5200 unit. Samples (100 mg) were heated to 140 °C at 10 °C min^{-1} and purged with Ar at 40 ml min^{-1} for 1 h before being cooled to room temperature. Subsequently the gas was

switched to 5% H_2/Ar at 40 ml min^{-1} before ramping from room temperature to 1000 °C at 10 °C min^{-1}, holding at 1000 °C for 15 min.

Co surface areas were determined for the five scaled-up samples by H_2 chemisorption at 150 °C in an ASAP 2010C Micromeritics apparatus by extrapolating the total gas uptakes in the H_2 adsorption isotherms at zero pressure. Samples (500 mg) were pre-treated in flowing He at 120 °C for 1 h before being reduced *in situ* by flowing H_2, raising the temperature to 425 °C and maintaining this temperature for 6 h.

The FTS reaction was carried out in a stainless-steel isothermal fixed bed six-way micro-reactor connected to an on-line VARIAN CP-3800 GC with three detectors. A Valco-valve was used to select between the gas streams for on-line analysis. The catalyst was diluted with SiC with a SiC to catalyst ratio of 3:1. Catalysts were reduced at 425 °C for 9 h in pure H_2 prior to testing. After reduction the reactor was cooled to 160 °C and H_2 was replaced with syngas (H_2:CO ratio of 2) and pressure was raised to 20 bar. The wax and water were collected from the gas-liquid separators and analysed using an off-line GC with a FID on a SimDist column.

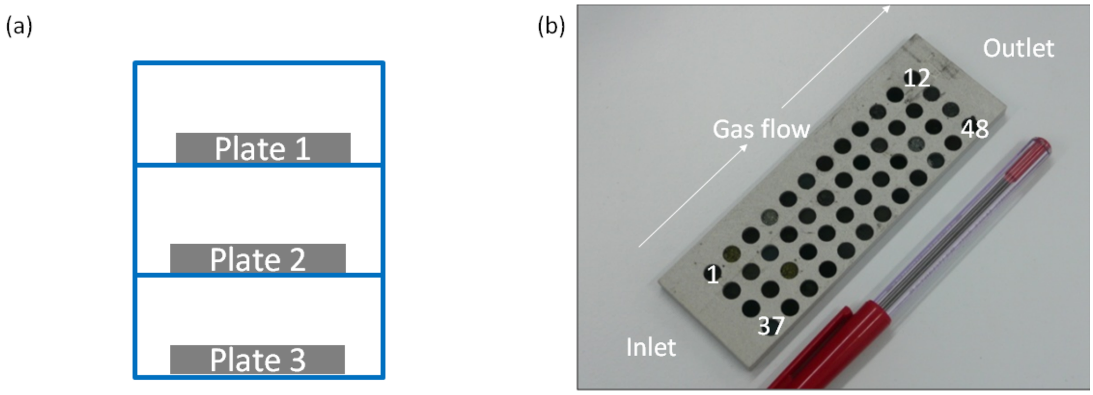

Supplementary figure S1. (a) End-on diagram of the stainless steel plate holder used to place 48-well plates in the furnace for gas treatments and (b) Image of the pyrophyllite 48-well plates used for gas treatments and HT XRD showing the direction of gas flow relative to the well numbering convention.

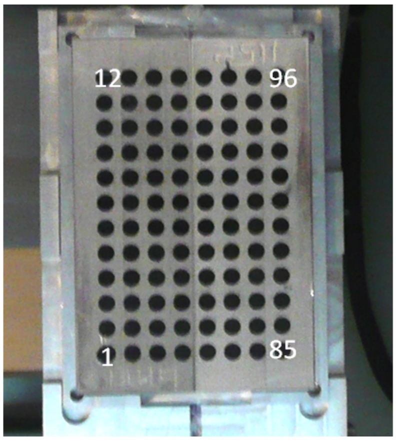

Supplementary figure S2. XYZ stage of the diffractometer with two 48-well plates in position. Note that the right hand side plate is rotated 180° with respect to the left hand plate. Numbering conventions are shown for the four corner wells.

(a)

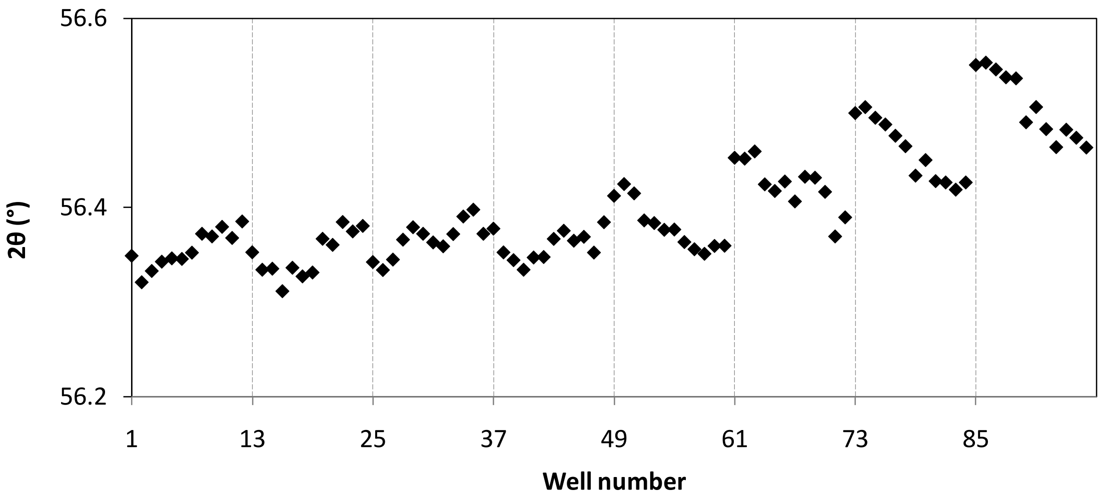

(b)

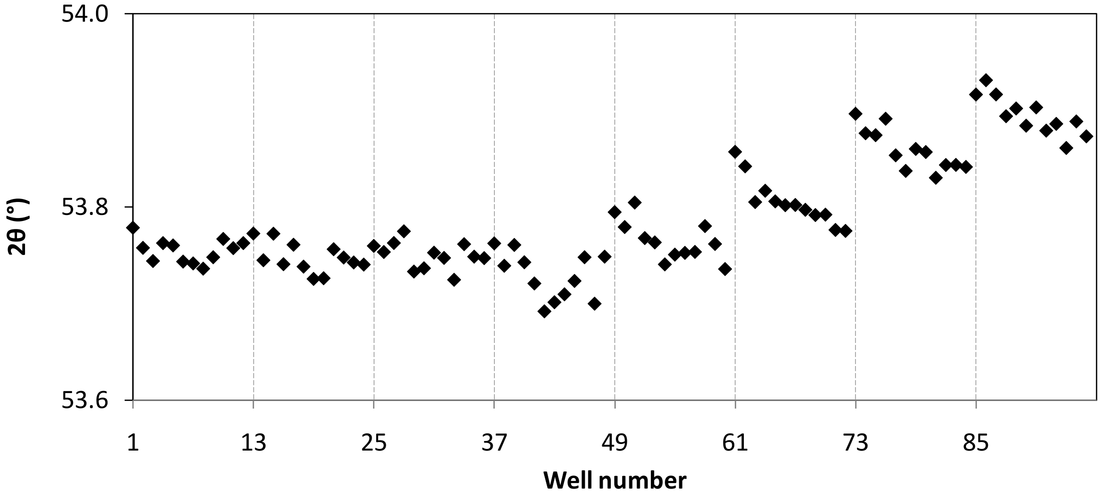

Supplementary figure S3. High-throughput XRD validation of position of a single peak of (a) TiO_2 and (b) Al_2O_3. Well number refers to well position on plate, A1 being number 1 and H12 being number 96.

(a)

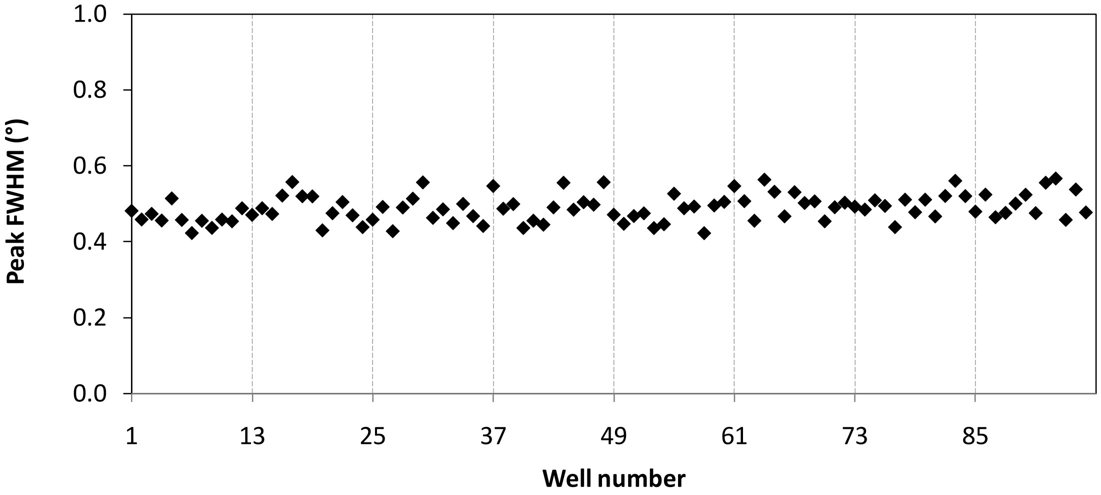

(b)

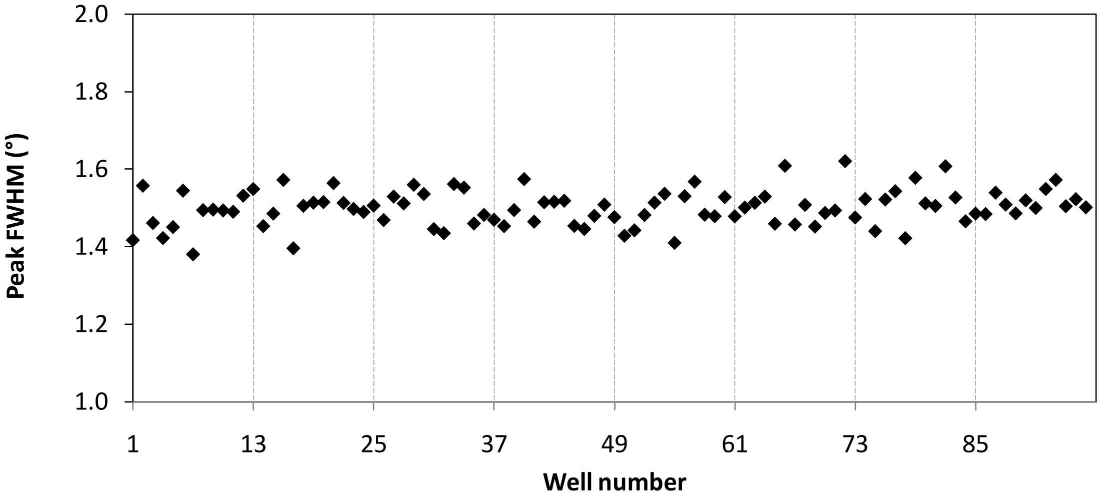

Supplementary figure S4. High-throughput XRD validation of FWHM of a single peak of (a) TiO_2 and (b) Al_2O_3. Well number refers to well position on plate, A1 being number 1 and H12 being number 96.

Supplementary table S1. Gas treatment validation showing mean cobalt particle sizes, normalised cobalt peak area and associated standard deviations pre- and post- ageing for each plate position in the furnace.

	Plate	Mean		Standard deviation	
		Co particle size (nm)	Normalised Co peak area	Co particle size (nm)	Normalised Co peak area
Pre ageing	Top	12.3	0.99	1.4	0.12
	Middle	12.4	1.01	1.5	0.13
	Bottom	12.4	1.04	1.5	0.12
	Overall	12.4	1.02	1.4	0.12
Post ageing	Top	13.6	0.43	1.5	0.11
	Middle	13.1	0.55	1.7	0.11
	Bottom	13.2	0.59	1.6	0.09
	Overall	13.3	0.52	1.6	0.12

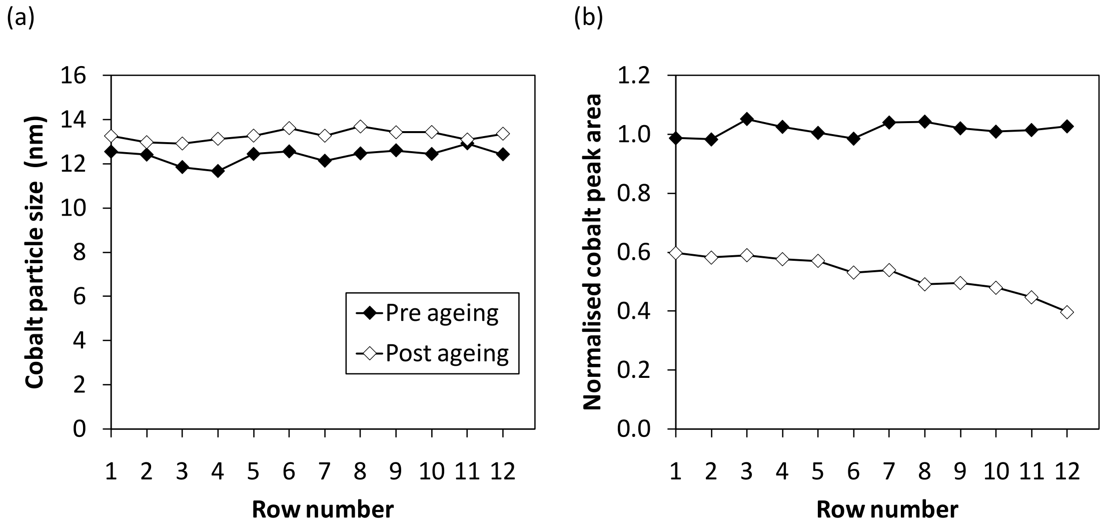

Supplementary figure S5. Gas treatment validation showing (a) mean cobalt particle sizes and (b) normalised cobalt peak area pre- and post- ageing treatment for each row position of plates. Row 1 is closest to the inlet and row 12 is closest to the outlet.

Supplementary table S2. Nominal loadings and impregnation volumes used to synthesise HT samples. Order of addition is defined in main text.

Loading wt %					1st impregnation					2nd impregnation					3rd impregnation			
Co	Mg	Ru	Order of addition		Soln	Volume of soln (mL)	Volume of water (mL)	Heat treatment temp		Soln	Volume of soln	Volume of water (mL)	Heat treatment temp (°C)		Soln	Volume of soln (mL)	Volume of water (mL)	Heat treatment temp (°C)
20	0.1	0.05	1		Mg	0.005	0.325	550		Co	0.305	0.025	120		Ru	0.053	0.277	250
20	0.1	0.1	1		Mg	0.005	0.325	550		Co	0.305	0.025	120		Ru	0.105	0.225	250
20	0.1	0.2	1		Mg	0.005	0.325	550		Co	0.305	0.025	120		Ru	0.211	0.119	250
20	0.5	0.05	1		Mg	0.025	0.305	550		Co	0.306	0.024	120		Ru	0.053	0.277	250
20	0.5	0.1	1		Mg	0.025	0.305	550		Co	0.306	0.024	120		Ru	0.106	0.224	250
20	0.5	0.2	1		Mg	0.025	0.305	550		Co	0.307	0.023	120		Ru	0.212	0.118	250
20	1	0.05	1		Mg	0.051	0.279	550		Co	0.308	0.022	120		Ru	0.053	0.277	250
20	1	0.1	1		Mg	0.051	0.279	550		Co	0.308	0.022	120		Ru	0.107	0.223	250
20	1	0.2	1		Mg	0.051	0.279	550		Co	0.309	0.021	120		Ru	0.214	0.116	250
20	2	0.05	1		Mg	0.103	0.227	550		Co	0.312	0.018	120		Ru	0.054	0.276	250
20	2	0.1	1		Mg	0.104	0.226	550		Co	0.312	0.018	120		Ru	0.108	0.222	250
20	2	0.2	1		Mg	0.104	0.226	550		Co	0.313	0.017	120		Ru	0.216	0.114	250
20	3	0.05	1		Mg	0.157	0.173	550		Co	0.316	0.014	120		Ru	0.055	0.275	250
20	3	0.1	1		Mg	0.157	0.173	550		Co	0.316	0.014	120		Ru	0.109	0.221	250
20	3	0.2	1		Mg	0.158	0.172	550		Co	0.317	0.013	120		Ru	0.219	0.111	250
20	4	0.05	1		Mg	0.212	0.118	550		Co	0.320	0.010	120		Ru	0.055	0.275	250
20	4	0.1	1		Mg	0.213	0.117	550		Co	0.321	0.009	120		Ru	0.111	0.219	250
20	4	0.2	1		Mg	0.213	0.117	550		Co	0.321	0.009	120		Ru	0.222	0.108	250
20	5	0.05	1		Mg	0.269	0.061	550		Co	0.325	0.005	120		Ru	0.056	0.274	250
20	5	0.1	1		Mg	0.269	0.061	550		Co	0.325	0.005	120		Ru	0.112	0.218	250
20	5	0.2	1		Mg	0.270	0.060	550		Co	0.325	0.005	120		Ru	0.225	0.105	250
20	6	0.05	1		Mg	0.327	0.003	550		Co	0.329	0.001	120		Ru	0.057	0.273	250
20	6	0.1	1		Mg	0.327	0.003	550		Co	0.329	0.001	120		Ru	0.114	0.216	250
20	6	0.2	1		Mg	0.328	0.002	550		Co	0.330	0.000	120		Ru	0.228	0.102	250
20	0.1	0.05	2		Mg	0.005	0.325	120		Co	0.305	0.025	120		Ru	0.053	0.277	250
20	0.1	0.1	2		Mg	0.005	0.325	120		Co	0.305	0.025	120		Ru	0.105	0.225	250
20	0.1	0.2	2		Mg	0.005	0.325	120		Co	0.305	0.025	120		Ru	0.211	0.119	250
20	0.5	0.05	2		Mg	0.025	0.305	120		Co	0.306	0.024	120		Ru	0.053	0.277	250
20	0.5	0.1	2		Mg	0.025	0.305	120		Co	0.306	0.024	120		Ru	0.106	0.224	250
20	0.5	0.2	2		Mg	0.025	0.305	120		Co	0.307	0.023	120		Ru	0.212	0.118	250
20	1	0.05	2		Mg	0.051	0.279	120		Co	0.308	0.022	120		Ru	0.053	0.277	250
20	1	0.1	2		Mg	0.051	0.279	120		Co	0.308	0.022	120		Ru	0.107	0.223	250
20	1	0.2	2		Mg	0.051	0.279	120		Co	0.309	0.021	120		Ru	0.214	0.116	250
20	2	0.05	2		Mg	0.103	0.227	120		Co	0.312	0.018	120		Ru	0.054	0.276	250

20	2	0.1	2		Mg	0.104	0.226	120		Co	0.312	0.018	120		Ru	0.108	0.222	250
20	2	0.2	2		Mg	0.104	0.226	120		Co	0.313	0.017	120		Ru	0.216	0.114	250
20	3	0.05	2		Mg	0.157	0.173	120		Co	0.316	0.014	120		Ru	0.055	0.275	250
20	3	0.1	2		Mg	0.157	0.173	120		Co	0.316	0.014	120		Ru	0.109	0.221	250
20	3	0.2	2		Mg	0.158	0.172	120		Co	0.317	0.013	120		Ru	0.219	0.111	250
20	4	0.05	2		Mg	0.212	0.118	120		Co	0.320	0.010	120		Ru	0.055	0.275	250
20	4	0.1	2		Mg	0.213	0.117	120		Co	0.321	0.009	120		Ru	0.111	0.219	250
20	4	0.2	2		Mg	0.213	0.117	120		Co	0.321	0.009	120		Ru	0.222	0.108	250
20	5	0.05	2		Mg	0.269	0.061	120		Co	0.325	0.005	120		Ru	0.056	0.274	250
20	5	0.1	2		Mg	0.269	0.061	120		Co	0.325	0.005	120		Ru	0.112	0.218	250
20	5	0.2	2		Mg	0.270	0.060	120		Co	0.325	0.005	120		Ru	0.225	0.105	250
20	6	0.05	2		Mg	0.327	0.003	120		Co	0.329	0.001	120		Ru	0.057	0.273	250
20	6	0.1	2		Mg	0.327	0.003	120		Co	0.329	0.001	120		Ru	0.114	0.216	250
20	6	0.2	2		Mg	0.328	0.002	120		Co	0.330	0.000	120		Ru	0.228	0.102	250
20	0.1	0.05	3		Co	0.329	0.001	120		Ru	0.057	0.273	120		Mg	0.005	0.325	250
20	0.1	0.1	3		Co	0.329	0.001	120		Ru	0.114	0.216	120		Mg	0.005	0.325	250
20	0.1	0.2	3		Co	0.329	0.001	120		Ru	0.228	0.102	120		Mg	0.005	0.325	250
20	0.5	0.05	3		Co	0.324	0.006	120		Ru	0.056	0.274	120		Mg	0.025	0.305	250
20	0.5	0.1	3		Co	0.306	0.024	120		Ru	0.106	0.224	120		Mg	0.025	0.305	250
20	0.5	0.2	3		Co	0.307	0.023	120		Ru	0.212	0.118	120		Mg	0.025	0.305	250
20	1	0.05	3		Co	0.308	0.022	120		Ru	0.053	0.277	120		Mg	0.051	0.279	250
20	1	0.1	3		Co	0.308	0.022	120		Ru	0.107	0.223	120		Mg	0.051	0.279	250
20	1	0.2	3		Co	0.308	0.022	120		Ru	0.214	0.116	120		Mg	0.051	0.279	250
20	2	0.05	3		Co	0.312	0.018	120		Ru	0.054	0.276	120		Mg	0.103	0.227	250
20	2	0.1	3		Co	0.312	0.018	120		Ru	0.108	0.222	120		Mg	0.104	0.226	250
20	2	0.2	3		Co	0.312	0.018	120		Ru	0.216	0.114	120		Mg	0.104	0.226	250
20	3	0.05	3		Co	0.316	0.014	120		Ru	0.055	0.275	120		Mg	0.157	0.173	250
20	3	0.1	3		Co	0.316	0.014	120		Ru	0.109	0.221	120		Mg	0.157	0.173	250
20	3	0.2	3		Co	0.316	0.014	120		Ru	0.219	0.111	120		Mg	0.158	0.172	250
20	4	0.05	3		Co	0.320	0.010	120		Ru	0.055	0.275	120		Mg	0.212	0.118	250
20	4	0.1	3		Co	0.320	0.010	120		Ru	0.111	0.219	120		Mg	0.213	0.117	250
20	4	0.2	3		Co	0.320	0.010	120		Ru	0.222	0.108	120		Mg	0.213	0.117	250
20	5	0.05	3		Co	0.305	0.025	120		Ru	0.053	0.277	120		Mg	0.269	0.061	250
20	5	0.1	3		Co	0.324	0.006	120		Ru	0.112	0.218	120		Mg	0.269	0.061	250
20	5	0.2	3		Co	0.324	0.006	120		Ru	0.225	0.105	120		Mg	0.270	0.060	250
20	6	0.05	3		Co	0.306	0.024	120		Ru	0.053	0.277	120		Mg	0.327	0.003	250
20	6	0.1	3		Co	0.305	0.025	120		Ru	0.105	0.225	120		Mg	0.327	0.003	250
20	6	0.2	3		Co	0.305	0.025	120		Ru	0.211	0.119	120		Mg	0.328	0.002	250

Supplementary table S3. Pre- ageing treatment data extracted from XRD data.

Synthesis parameters (wt%)					Cobalt peak					Support peak						
Co	Mg	Ru	Order of addition		2theta (°)	FWHM (°)	Area (cts*°)	Intensity (cts)		2theta (°)	FWHM (°)	Area (cts*°)	Intensity (cts)		PS (nm)	Normalised Co area
20	0.1	0.05	1		52.07	0.86	62.60	86.32		54.07	1.32	60.71	55.32		11.95	1.13
20	0.1	0.05	1		52.09	0.79	60.89	88.57		53.82	1.60	91.02	67.89		13.06	0.90
20	0.1	0.1	1		51.93	0.85	73.79	102.92		53.83	1.19	71.29	71.93		12.12	1.03
20	0.1	0.1	1		51.97	0.78	77.71	120.08		53.91	1.54	95.48	74.61		13.23	1.04
20	0.1	0.2	1		51.94	0.90	66.11	85.00		54.03	1.24	64.71	64.08		11.46	1.03
20	0.1	0.2	1		51.97	0.84	58.94	85.54		53.97	1.61	69.69	51.94		12.26	1.13
20	0.5	0.05	1		51.91	0.93	99.49	123.23		53.85	1.24	89.92	88.22		11.02	1.13
20	0.5	0.05	1		51.95	1.26	118.47	110.06		54.09	1.42	77.52	71.37		8.12	1.66
20	0.5	0.1	1		51.92	1.01	104.09	123.04		53.90	1.39	94.54	82.46		10.11	1.26
20	0.5	0.1	1		52.01	0.97	110.04	140.13		54.01	1.73	137.34	96.67		10.61	1.14
20	0.5	0.2	1		51.98	1.01	72.10	86.24		53.91	1.46	74.31	61.68		10.13	1.17
20	0.5	0.2	1		51.98	1.02	92.67	105.36		53.99	1.32	80.92	75.95		10.08	1.22
20	1	0.05	1		51.89	0.93	90.94	116.30		53.77	1.34	68.41	62.41		11.03	1.46
20	1	0.05	1		51.97	0.90	107.44	141.90		53.92	1.38	81.36	71.86		11.44	1.50
20	1	0.1	1		51.98	1.12	77.86	82.38		53.98	1.15	59.01	62.13		9.13	1.25
20	1	0.1	1		52.04	1.07	87.46	94.59		54.06	1.36	65.99	60.23		9.56	1.45
20	1	0.2	1		51.96	1.02	89.58	101.08		53.92	1.22	73.89	74.02		10.02	1.21
20	1	0.2	1		51.83	0.96	78.40	97.89		53.75	1.31	81.14	73.68		10.69	1.06
20	2	0.05	1		51.94	0.99	82.41	99.19		53.77	1.32	63.03	58.73		10.32	1.40
20	2	0.05	1		51.96	1.02	103.94	118.05		53.85	1.15	74.86	81.30		10.07	1.28
20	2	0.1	1		51.81	0.92	104.91	135.50		53.67	1.29	89.57	84.39		11.12	1.24
20	2	0.1	1		51.89	1.09	114.13	121.90		53.83	1.16	76.07	80.68		9.44	1.41
20	2	0.2	1		51.94	0.95	66.43	83.11		53.80	1.31	50.44	47.20		10.79	1.41
20	2	0.2	1		51.94	0.93	76.27	97.73		53.78	1.26	66.23	63.90		11.03	1.19
20	3	0.05	1		51.95	1.15	104.56	109.20		53.73	1.18	75.53	77.38		8.96	1.35
20	3	0.05	1		52.02	1.20	82.43	83.70		53.83	1.43	66.78	59.46		8.53	1.39
20	3	0.1	1		51.97	1.34	116.56	101.93		53.98	0.83	42.86	81.19		7.69	1.44
20	3	0.1	1		51.92	1.10	111.35	122.05		53.71	1.31	100.05	92.26		9.31	1.21
20	3	0.2	1		51.81	0.96	94.04	118.65		53.57	1.32	97.33	89.60		10.68	1.05

20	3	0.2	1		51.95	0.97	120.12	150.41		53.71	1.34	124.15	112.45		10.59	1.07
20	4	0.05	1		51.97	1.03	135.24	157.87		53.77	1.32	103.33	96.14		9.97	1.41
20	4	0.05	1		51.83	0.94	82.96	105.92		53.60	1.33	77.20	70.51		10.87	1.18
20	4	0.1	1		51.86	1.09	81.25	90.91		53.65	1.26	66.78	64.41		9.40	1.26
20	4	0.1	1		51.85	1.14	113.95	120.16		53.69	1.33	72.34	68.47		9.00	1.66
20	4	0.2	1		51.96	1.39	143.16	120.24		53.85	1.14	78.10	94.09		7.38	1.52
20	4	0.2	1		51.98	1.14	125.87	131.93		53.70	1.14	80.96	88.99		8.99	1.41
20	5	0.05	1		52.01	1.38	120.18	101.84		53.83	1.10	68.13	84.64		7.45	1.42
20	5	0.05	1		51.98	1.11	102.88	113.53		53.74	1.33	102.95	94.68		9.25	1.09
20	5	0.1	1		52.01	0.99	74.58	90.80		53.77	1.27	67.61	64.82		10.38	1.15
20	5	0.1	1		51.96	1.24	106.33	103.27		53.75	1.22	76.55	78.69		8.26	1.35
20	5	0.2	1		51.92	1.21	89.57	90.96		53.70	1.34	79.79	73.83		8.51	1.21
20	5	0.2	1		51.87	1.14	91.82	98.46		53.72	1.40	88.48	77.82		9.00	1.18
20	6	0.05	1		51.87	1.13	172.14	187.93		53.62	1.41	162.00	142.74		9.11	1.21
20	6	0.05	1		51.84	0.97	87.11	109.49		53.58	1.29	94.53	88.73		10.62	0.98
20	6	0.1	1		51.83	1.38	118.19	103.36		53.58	1.18	86.32	89.20		7.41	1.33
20	6	0.1	1		51.84	1.63	115.84	83.55		53.60	1.48	93.79	76.44		6.31	1.52
20	6	0.2	1		51.86	1.22	104.14	102.95		53.71	1.24	87.39	86.79		8.38	1.20
20	6	0.2	1		51.86	1.17	85.79	88.24		53.72	1.14	66.65	71.34		8.80	1.20
20	0.1	0.05	2		51.92	0.80	86.09	127.33		53.83	1.56	87.83	68.06		12.79	1.26
20	0.1	0.05	2		51.92	0.88	75.98	103.38		53.81	1.51	67.67	54.83		11.66	1.39
20	0.1	0.1	2		51.87	0.98	125.23	148.41		53.83	1.30	112.17	104.61		10.51	1.20
20	0.1	0.1	2		51.84	0.83	70.04	102.43		53.78	1.63	88.09	65.03		12.34	1.08
20	0.1	0.2	2		51.83	0.93	87.48	116.68		53.84	1.63	97.27	71.93		11.07	1.22
20	0.1	0.2	2		51.86	1.03	90.75	104.62		53.87	1.23	72.48	71.41		9.94	1.27
20	0.5	0.05	2		51.81	1.02	78.36	92.00		53.71	1.41	72.48	62.16		10.03	1.26
20	0.5	0.05	2		51.80	1.15	93.68	94.91		53.86	1.19	59.21	62.67		8.90	1.49
20	0.5	0.1	2		51.87	0.87	105.82	147.60		53.76	1.57	111.48	85.71		11.77	1.23
20	0.5	0.1	2		51.77	1.05	125.77	144.64		53.76	1.46	108.74	91.06		9.80	1.38
20	0.5	0.2	2		51.89	0.89	78.03	104.01		53.86	1.32	63.93	58.40		11.56	1.34
20	0.5	0.2	2		51.86	0.95	72.99	92.06		53.79	1.37	66.36	58.73		10.81	1.24
20	1	0.05	2		51.83	1.05	92.69	104.06		53.95	1.20	60.05	62.45		9.81	1.48
20	1	0.05	2		51.84	0.86	84.87	119.39		53.83	1.74	92.00	64.15		11.89	1.32
20	1	0.1	2		51.88	0.91	68.55	88.69		53.78	1.12	48.91	52.94		11.28	1.29

20	1	0.1	2		51.81	0.99	124.66	150.67		53.82	1.49	113.95	93.26		10.31	1.34
20	1	0.2	2		51.82	1.15	132.30	139.36		53.83	1.49	106.91	88.21		8.94	1.50
20	1	0.2	2		51.81	1.17	91.73	93.52		53.87	1.22	66.29	66.35		8.77	1.38
20	2	0.05	2		51.91	0.99	95.56	115.24		53.82	1.44	66.99	57.29		10.39	1.67
20	2	0.05	2		51.83	1.05	119.30	133.64		53.76	1.05	74.82	86.72		9.73	1.38
20	2	0.1	2		51.86	0.81	79.38	115.45		53.72	1.39	61.10	53.67		12.63	1.48
20	2	0.1	2		51.78	0.86	141.07	197.11		53.66	1.48	135.37	111.33		11.96	1.27
20	2	0.2	2		51.82	0.89	73.72	97.88		53.79	1.29	59.10	55.38		11.53	1.33
20	2	0.2	2		51.75	0.94	115.06	145.91		53.67	1.37	100.68	89.58		10.89	1.28
20	3	0.05	2		51.83	0.81	80.43	119.85		53.68	1.61	87.69	67.32		12.60	1.19
20	3	0.05	2		51.78	0.99	78.91	96.20		53.69	1.51	64.54	52.38		10.38	1.51
20	3	0.1	2		51.84	1.02	105.61	127.12		53.76	1.73	96.07	68.93		10.08	1.53
20	3	0.1	2		51.83	0.94	41.08	51.45		53.61	1.01	22.78	27.21		10.96	1.51
20	3	0.2	2		51.81	1.20	141.20	137.52		53.61	1.37	111.04	98.05		8.58	1.44
20	3	0.2	2		51.86	1.06	82.63	92.35		53.64	1.08	57.69	65.62		9.64	1.26
20	4	0.05	2		51.80	1.07	133.52	150.23		53.66	1.35	94.84	86.68		9.63	1.54
20	4	0.05	2		51.75	1.02	130.95	148.72		53.58	1.24	98.81	98.15		10.02	1.33
20	4	0.1	2		51.84	0.89	81.78	110.62		53.63	1.59	78.72	60.50		11.48	1.35
20	4	0.1	2		51.78	0.84	97.76	138.47		53.64	1.43	92.85	78.70		12.15	1.24
20	4	0.2	2		51.79	1.09	110.21	124.80		53.59	1.65	107.57	79.56		9.40	1.39
20	4	0.2	2		51.82	1.12	94.80	98.76		53.65	1.17	66.20	70.72		9.19	1.34
20	5	0.05	2		51.90	0.84	51.32	73.85		53.62	1.36	53.73	47.85		12.27	1.07
20	5	0.05	2		51.81	0.97	72.28	89.15		53.66	1.25	60.80	59.09		10.60	1.22
20	5	0.1	2		51.81	1.10	84.15	90.98		53.67	1.36	61.52	55.20		9.35	1.52
20	5	0.1	2		51.81	1.16	112.70	115.90		53.71	1.16	69.44	74.10		8.86	1.52
20	5	0.2	2		51.82	0.93	63.92	81.89		53.62	1.27	57.53	55.15		10.99	1.16
20	5	0.2	2		51.78	0.95	80.63	101.23		53.48	1.18	55.56	57.96		10.83	1.39
20	6	0.05	2		51.76	1.17	176.09	181.54		53.60	1.28	144.98	139.16		8.75	1.27
20	6	0.05	2		51.75	0.95	78.66	100.51		53.47	1.36	73.02	65.57		10.82	1.20
20	6	0.1	2		51.80	1.11	115.51	125.27		53.56	1.29	88.07	84.72		9.21	1.36
20	6	0.1	2		51.82	1.06	74.07	83.42		53.65	1.10	59.80	65.82		9.68	1.13
20	6	0.2	2		51.82	1.28	96.73	89.83		53.84	1.12	48.87	54.67		8.03	1.77
20	6	0.2	2		51.83	1.03	115.77	131.85		53.75	1.14	73.95	79.77		10.00	1.45
20	0.1	0.05	3		51.97	0.69	98.07	165.14		54.03	1.41	72.51	61.86		14.92	1.59

20	0.1	0.05	3		51.89	0.73	127.89	205.03		53.79	1.27	93.57	89.13		14.13	1.43
20	0.1	0.1	3		51.83	0.88	63.51	85.38		53.87	1.34	46.66	42.30		11.71	1.50
20	0.1	0.1	3		51.88	0.75	123.57	191.05		53.84	1.36	84.23	75.15		13.61	1.64
20	0.1	0.2	3		51.85	0.82	114.85	159.99		53.76	1.18	78.20	80.56		12.46	1.43
20	0.1	0.2	3		51.88	0.78	114.29	172.75		53.84	1.64	113.80	83.88		13.15	1.36
20	0.5	0.05	3		51.85	0.71	138.34	228.39		53.63	2.50	160.02	78.80		14.41	1.76
20	0.5	0.05	3		51.91	0.82	115.30	161.07		53.83	1.62	87.94	66.94		12.50	1.72
20	0.5	0.1	3		51.87	0.74	95.98	156.50		53.78	1.72	99.08	70.00		13.90	1.37
20	0.5	0.1	3		51.97	0.85	106.73	144.60		53.97	1.30	73.47	68.88		12.10	1.55
20	0.5	0.2	3		51.93	0.89	101.76	133.67		54.08	0.90	50.06	70.65		11.48	1.44
20	0.5	0.2	3		51.84	0.87	202.31	277.13		53.79	1.46	178.53	148.04		11.80	1.37
20	1	0.05	3		51.88	0.86	105.01	143.43		53.84	1.32	60.95	56.82		11.97	1.85
20	1	0.05	3		51.96	0.85	104.88	148.43		53.78	1.72	79.84	57.65		12.07	1.82
20	1	0.1	3		51.85	0.83	132.05	188.59		53.77	1.36	120.47	107.03		12.41	1.23
20	1	0.1	3		51.95	0.77	108.28	166.34		53.91	1.51	88.59	71.00		13.39	1.53
20	1	0.2	3		51.89	0.80	119.54	174.98		53.79	1.26	85.58	82.26		12.82	1.45
20	1	0.2	3		51.88	0.71	60.88	100.27		53.87	1.57	59.47	45.58		14.36	1.34
20	2	0.05	3		51.86	0.80	106.29	163.43		53.69	1.69	119.92	85.76		12.83	1.24
20	2	0.05	3		51.91	0.85	123.42	169.72		53.86	1.24	87.34	85.14		12.02	1.45
20	2	0.1	3		51.82	0.75	110.40	172.36		53.71	1.39	92.32	80.15		13.63	1.38
20	2	0.1	3		51.86	0.81	113.21	174.84		53.70	1.99	136.99	83.92		12.73	1.35
20	2	0.2	3		51.91	0.76	108.47	166.88		53.77	1.20	73.33	73.97		13.55	1.47
20	2	0.2	3		51.89	0.84	184.78	249.30		53.81	1.24	137.96	138.27		12.19	1.34
20	3	0.05	3		51.86	0.79	106.03	158.91		53.73	1.42	95.49	81.38		12.99	1.30
20	3	0.05	3		51.91	0.71	86.53	142.44		53.59	1.33	66.84	61.32		14.38	1.41
20	3	0.1	3		51.87	0.74	103.57	165.56		53.69	1.36	95.22	84.19		13.92	1.23
20	3	0.1	3		51.85	0.84	122.14	173.91		53.72	1.59	113.79	87.16		12.15	1.40
20	3	0.2	3		51.82	0.81	113.36	163.04		53.69	1.19	79.40	80.71		12.60	1.40
20	3	0.2	3		51.91	0.83	107.18	152.51		53.77	1.33	74.80	68.99		12.41	1.55
20	4	0.05	3		51.88	0.81	91.01	133.11		53.68	1.40	77.60	67.44		12.59	1.35
20	4	0.05	3		51.83	0.99	142.37	166.39		53.67	1.19	87.68	91.22		10.36	1.56
20	4	0.1	3		51.86	0.95	148.44	181.03		53.58	1.44	114.67	96.48		10.84	1.54
20	4	0.1	3		51.87	0.85	88.95	126.68		53.58	1.42	70.45	60.49		12.08	1.47
20	4	0.2	3		51.86	0.91	116.05	150.88		53.70	1.26	95.29	91.67		11.22	1.27

20	4	0.2	3		51.81	1.02	194.00	220.99		53.67	1.29	134.17	130.24		10.09	1.49
20	5	0.05	3		51.86	0.95	135.34	169.55		53.63	1.29	100.48	95.72		10.76	1.41
20	5	0.05	3		51.96	0.81	86.38	127.54		53.66	1.40	83.42	72.26		12.62	1.20
20	5	0.1	3		51.90	0.85	106.09	155.03		53.59	1.68	124.12	90.41		12.05	1.17
20	5	0.1	3		51.89	0.86	103.11	141.36		53.70	1.24	78.21	76.87		11.90	1.34
20	5	0.2	3		51.82	0.78	116.86	185.17		53.57	1.76	135.41	93.93		13.21	1.24
20	5	0.2	3		51.90	0.88	133.66	175.10		53.69	1.32	104.42	97.09		11.67	1.38
20	6	0.05	3		51.84	0.89	109.30	146.50		53.62	1.25	88.21	85.87		11.57	1.27
20	6	0.05	3		51.90	0.78	97.57	151.85		53.61	1.55	112.74	87.99		13.11	1.11
20	6	0.1	3		51.82	0.87	100.75	138.51		53.52	1.30	92.14	86.18		11.76	1.17
20	6	0.1	3		51.88	0.78	83.89	123.30		53.42	1.37	72.85	61.96		13.20	1.35
20	6	0.2	3		51.84	0.89	126.44	168.93		53.61	1.32	95.73	89.07		11.51	1.42
20	6	0.2	3		51.90	0.96	160.55	200.24		53.63	1.38	115.56	104.36		10.66	1.54

Supplementary table S4. Post-ageing treatment data extracted from XRD data.

Synthesis parameters (wt%)					Cobalt peak					Support peak						
Co	Mg	Ru	Order of addition		2theta (°)	FWHM (°)	Area (cts*°)	Intensity (cts)		2theta (°)	FWHM (°)	Area (cts*°)	Intensity (cts)		PS (nm)	Normalised Co area
20	0.1	0.05	1		51.87	0.79	67.54	102.67		53.83	1.42	96.32	80.82		13.01	0.84
20	0.1	0.05	1		52.07	0.67	49.15	87.30		53.94	1.41	82.06	69.34		15.28	0.71
20	0.1	0.1	1		51.92	0.71	95.68	158.24		53.85	1.25	117.51	111.84		14.46	0.86
20	0.1	0.1	1		51.91	0.70	66.71	111.41		53.86	1.31	83.92	76.30		14.59	0.87
20	0.1	0.2	1		51.89	0.77	68.94	106.24		53.90	1.28	88.41	82.13		13.38	0.84
20	0.1	0.2	1		51.92	0.78	65.27	99.90		53.91	1.37	83.57	72.78		13.23	0.90
20	0.5	0.05	1		52.00	0.88	54.95	74.22		53.87	1.20	61.30	61.14		11.63	0.90
20	0.5	0.05	1		51.94	0.82	65.86	95.55		53.89	1.34	80.91	72.39		12.49	0.91
20	0.5	0.1	1		52.04	1.03	93.92	109.49		54.01	1.18	100.53	101.94		10.00	0.92
20	0.5	0.1	1		51.95	0.96	104.30	129.48		53.92	1.26	115.18	109.41		10.64	0.95
20	0.5	0.2	1		51.90	0.88	86.74	124.25		53.89	1.81	153.11	101.45		11.65	0.86
20	0.5	0.2	1		51.96	0.81	77.89	110.20		53.97	1.09	82.61	92.86		12.68	0.84
20	1	0.05	1		52.02	0.71	78.14	134.70		53.83	1.53	135.94	106.39		14.49	0.73
20	1	0.05	1		51.94	0.69	75.37	129.07		53.79	1.34	96.38	85.58		14.87	0.88

20	1	0.1	1		52.02	1.05	157.95	174.48		54.02	1.11	125.25	149.49		9.78	1.06
20	1	0.1	1		51.98	0.92	71.34	93.85		53.86	1.28	90.37	84.35		11.21	0.85
20	1	0.2	1		51.82	1.16	99.89	102.57		53.76	1.07	77.29	87.81		8.83	1.14
20	1	0.2	1		51.89	1.48	125.99	103.09		53.84	1.12	97.39	108.40		6.95	1.16
20	2	0.05	1		51.98	1.11	82.55	86.60		53.84	1.09	68.00	75.97		9.27	1.09
20	2	0.05	1		51.91	0.98	84.87	105.02		53.74	1.35	97.42	87.54		10.45	0.97
20	2	0.1	1		51.89	0.87	111.58	146.96		53.70	1.30	106.29	98.08		11.74	1.14
20	2	0.1	1		51.89	0.83	118.75	171.75		53.67	1.32	120.03	109.35		12.42	1.09
20	2	0.2	1		51.98	0.99	96.46	117.47		53.82	1.34	93.44	84.69		10.36	1.14
20	2	0.2	1		51.93	1.27	134.39	123.86		53.91	1.17	86.78	93.04		8.11	1.44
20	3	0.05	1		51.99	1.09	101.20	111.36		53.85	1.19	91.72	93.72		9.40	1.08
20	3	0.05	1		51.90	1.00	77.09	94.38		53.72	1.39	86.05	74.97		10.30	1.03
20	3	0.1	1		51.99	0.86	93.94	125.41		53.68	1.41	110.19	93.53		11.92	1.00
20	3	0.1	1		51.99	0.99	78.06	95.94		53.73	1.31	99.78	91.91		10.39	0.85
20	3	0.2	1		52.00	1.16	138.92	146.53		53.75	1.19	146.51	149.83		8.87	0.93
20	3	0.2	1		51.86	1.09	132.55	150.77		53.62	1.43	140.74	120.92		9.45	1.10
20	4	0.05	1		51.89	0.74	95.15	154.83		53.53	1.41	116.47	99.22		13.83	0.96
20	4	0.05	1		51.95	0.89	154.33	200.31		53.72	1.15	137.94	146.82		11.56	1.05
20	4	0.1	1		51.93	0.96	108.90	137.27		53.63	1.23	112.29	110.93		10.70	0.98
20	4	0.1	1		51.87	1.04	105.02	122.57		53.63	1.27	105.72	101.42		9.86	1.04
20	4	0.2	1		52.01	1.05	99.35	117.89		53.75	1.48	115.91	95.93		9.77	1.04
20	4	0.2	1		51.96	1.18	120.32	118.93		53.75	1.12	104.42	123.98		8.72	0.97
20	5	0.05	1		52.04	1.27	103.41	104.69		53.73	1.25	173.80	169.18		8.09	0.61
20	5	0.05	1		52.04	1.54	114.36	86.84		53.70	1.24	94.19	98.66		6.66	1.16
20	5	0.1	1		51.98	0.97	95.43	121.05		53.68	1.46	138.72	114.81		10.53	0.83
20	5	0.1	1		52.04	1.15	96.16	104.13		53.76	1.24	132.92	129.53		8.92	0.74
20	5	0.2	1		51.98	1.32	129.05	120.59		53.72	1.17	149.54	157.20		7.75	0.82
20	5	0.2	1		51.94	1.28	93.24	92.70		53.64	1.35	126.16	114.79		7.99	0.81
20	6	0.05	1		51.81	0.85	107.64	159.74		53.52	1.61	181.42	135.40		12.02	0.79
20	6	0.05	1		51.94	1.08	136.27	145.83		53.74	0.99	88.55	131.42		9.46	1.04
20	6	0.1	1		52.00	1.15	107.90	114.05		53.70	1.06	121.97	138.79		8.91	0.78
20	6	0.1	1		51.93	1.17	84.49	90.99		53.55	1.24	109.54	107.75		8.80	0.78
20	6	0.2	1		52.01	1.30	137.03	128.62		53.71	1.12	125.18	139.46		7.89	0.98
20	6	0.2	1		51.97	1.67	131.15	96.92		53.70	1.16	102.31	119.80		6.14	1.09

20	0.1	0.05	2		51.98	0.64	58.86	109.13		53.82	1.44	102.86	84.85		15.92	0.69
20	0.1	0.05	2		51.99	0.73	61.55	99.79		53.85	1.25	77.07	73.43		14.09	0.84
20	0.1	0.1	2		51.98	0.84	71.47	101.14		53.89	1.33	83.93	75.35		12.17	0.95
20	0.1	0.1	2		52.02	1.09	67.98	72.12		53.97	1.39	74.52	64.74		9.41	1.05
20	0.1	0.2	2		51.97	0.86	69.75	96.85		53.95	1.32	91.61	82.97		11.94	0.84
20	0.1	0.2	2		52.09	1.10	74.17	78.46		54.10	1.12	59.10	68.10		9.36	1.09
20	0.5	0.05	2		51.94	0.82	44.63	64.45		53.92	1.18	58.82	58.99		12.49	0.76
20	0.5	0.05	2		51.99	0.86	49.28	68.75		53.99	1.26	60.45	65.38		11.97	0.75
20	0.5	0.1	2		51.83	1.17	131.37	134.43		53.86	1.18	113.03	116.42		8.80	1.13
20	0.5	0.1	2		51.97	1.18	79.85	80.58		54.02	1.19	68.49	69.88		8.67	1.14
20	0.5	0.2	2		51.90	1.05	70.55	80.90		53.83	1.17	80.38	81.99		9.78	0.86
20	0.5	0.2	2		52.00	0.95	59.52	75.39		53.91	1.35	72.36	64.47		10.75	0.92
20	1	0.05	2		51.96	0.98	78.03	91.77		53.84	1.27	86.16	82.18		10.44	0.95
20	1	0.05	2		51.96	0.82	107.94	157.39		54.00	1.06	143.85	164.36		12.51	0.66
20	1	0.1	2		51.95	0.90	94.02	120.07		53.94	1.13	103.14	113.85		11.37	0.83
20	1	0.1	2		51.99	1.03	63.85	74.32		53.89	1.15	68.92	72.11		9.96	0.89
20	1	0.2	2		51.91	0.80	52.74	78.38		53.87	1.27	72.20	67.56		12.85	0.78
20	1	0.2	2		51.95	0.80	71.14	108.50		53.88	1.32	90.78	82.85		12.85	0.86
20	2	0.05	2		51.93	0.96	76.98	96.88		53.83	1.50	74.99	61.07		10.69	1.26
20	2	0.05	2		52.03	1.05	104.65	118.74		53.98	1.27	86.67	82.68		9.75	1.27
20	2	0.1	2		51.87	0.98	131.00	159.03		53.77	1.32	106.31	98.36		10.51	1.33
20	2	0.1	2		52.00	0.84	84.59	115.50		53.88	1.65	90.75	66.88		12.20	1.26
20	2	0.2	2		51.93	0.82	97.86	139.78		54.15	0.00	39.01	18.55		12.50	5.27*
20	2	0.2	2		51.98	0.82	85.42	124.21		53.85	1.40	94.52	81.04		12.48	1.05
20	3	0.05	2		51.90	0.92	62.37	102.74		53.66	1.32	57.57	54.35		11.09	1.15
20	3	0.05	2		51.94	0.82	124.98	182.14		53.77	1.38	133.82	117.66		12.51	1.06
20	3	0.1	2		51.87	0.99	115.03	140.72		53.78	1.38	116.94	102.34		10.41	1.12
20	3	0.1	2		51.96	0.95	98.93	125.46		53.84	1.37	92.75	82.18		10.85	1.20
20	3	0.2	2		51.92	0.93	71.24	90.56		53.73	1.21	58.57	58.72		11.05	1.21
20	3	0.2	2		51.95	0.94	94.15	116.16		53.89	1.10	72.82	82.19		10.97	1.15
20	4	0.05	2		51.88	0.93	129.43	166.11		53.70	1.25	130.68	126.19		10.98	1.03
20	4	0.05	2		51.93	0.93	59.50	78.51		53.73	1.49	62.79	50.96		11.00	1.17
20	4	0.1	2		51.90	0.91	78.53	103.06		53.70	1.24	67.61	66.14		11.31	1.19
20	4	0.1	2		51.92	0.83	65.41	93.33		53.76	1.29	69.57	64.59		12.29	1.01

20	4	0.2	2		51.89	0.88	55.70	72.75		53.69	1.16	52.60	55.11		11.64	1.01
20	4	0.2	2		51.98	0.84	76.61	108.26		53.75	1.23	77.14	75.27		12.18	1.02
20	5	0.05	2		51.88	0.74	74.60	121.06		53.63	1.52	91.95	72.80		13.77	1.02
20	5	0.05	2		51.95	0.79	43.13	65.17		53.71	1.34	52.31	46.87		12.92	0.92
20	5	0.1	2		51.92	0.77	73.58	113.71		53.76	1.40	89.96	77.23		13.26	0.95
20	5	0.1	2		52.01	1.02	74.18	83.83		53.79	1.19	60.52	61.99		10.02	1.20
20	5	0.2	2		51.86	0.80	55.15	86.57		53.51	1.47	73.77	60.37		12.83	0.91
20	5	0.2	2		51.96	0.82	75.02	108.45		53.70	1.20	66.00	66.47		12.54	1.13
20	6	0.05	2		51.93	0.87	69.73	94.45		53.71	1.07	54.99	61.89		11.80	1.13
20	6	0.05	2		51.79	0.91	134.67	187.96		53.57	1.76	185.91	128.23		11.29	1.05
20	6	0.1	2		51.92	0.93	60.55	77.36		53.66	1.38	43.30	38.85		11.06	1.56
20	6	0.1	2		51.96	0.95	92.48	112.88		53.76	1.19	81.16	85.07		10.85	1.09
20	6	0.2	2		52.03	1.00	75.58	89.71		53.94	1.16	61.01	63.55		10.26	1.19
20	6	0.2	2		51.96	1.27	107.14	98.23		53.93	1.18	64.86	70.09		8.07	1.53
20	0.1	0.05	3		51.92	0.71	137.60	230.58		53.85	1.80	148.66	100.29		14.35	1.37
20	0.1	0.05	3		51.94	0.63	128.60	238.60		53.85	1.68	130.19	93.69		16.21	1.37
20	0.1	0.1	3		51.94	0.64	84.16	153.02		53.86	1.50	77.65	62.47		16.04	1.35
20	0.1	0.1	3		51.94	0.66	71.57	125.33		53.94	1.23	55.97	54.24		15.59	1.32
20	0.1	0.2	3		51.94	0.64	77.85	140.03		53.87	1.31	67.46	61.54		15.97	1.27
20	0.1	0.2	3		51.93	0.71	97.81	159.51		53.86	1.30	77.79	72.49		14.37	1.35
20	0.5	0.05	3		51.91	0.67	115.70	200.68		53.80	1.52	118.57	93.65		15.22	1.24
20	0.5	0.05	3		51.96	0.67	71.40	121.81		54.00	1.68	70.71	51.30		15.22	1.39
20	0.5	0.1	3		51.97	0.63	94.53	175.56		53.88	1.52	97.31	76.86		16.34	1.23
20	0.5	0.1	3		51.96	0.64	76.26	139.54		53.90	1.59	85.35	64.58		16.03	1.18
20	0.5	0.2	3		51.89	0.79	253.45	365.43		53.83	1.75	263.30	176.55		12.92	1.44
20	0.5	0.2	3		51.93	0.69	89.59	153.20		53.86	1.52	104.21	82.46		14.87	1.09
20	1	0.05	3		51.94	0.61	119.46	226.89		53.86	1.46	107.20	88.47		16.87	1.35
20	1	0.05	3		51.91	0.64	109.31	198.71		53.73	1.54	86.54	68.48		16.02	1.60
20	1	0.1	3		51.87	0.79	158.84	233.66		53.78	1.53	141.71	112.24		13.04	1.42
20	1	0.1	3		51.86	0.96	206.17	248.50		53.95	0.99	88.16	143.30		10.71	1.44
20	1	0.2	3		51.91	0.65	95.23	169.34		53.76	1.43	78.31	66.49		15.68	1.43
20	1	0.2	3		51.93	0.63	101.36	185.99		53.84	1.49	95.84	77.41		16.19	1.31
20	2	0.05	3		51.94	0.73	118.62	190.71		53.80	1.37	97.88	90.31		14.11	1.31
20	2	0.05	3		51.89	0.64	80.52	147.88		53.73	1.64	80.70	59.42		15.99	1.36

20	2	0.1	3		51.88	0.56	75.23	153.38		53.63	1.48	57.60	47.16		18.24	1.60
20	2	0.1	3		51.90	0.64	87.80	160.07		53.76	1.66	80.34	59.03		15.96	1.49
20	2	0.2	3		51.91	0.70	114.52	189.54		53.84	1.25	81.11	78.40		14.73	1.46
20	2	0.2	3		51.91	0.65	119.84	212.31		53.77	1.30	90.36	83.71		15.74	1.43
20	3	0.05	3		51.93	0.68	110.10	192.52		53.70	1.65	99.41	73.60		15.20	1.50
20	3	0.05	3		51.97	0.68	94.14	163.39		53.77	1.47	81.41	66.93		15.18	1.41
20	3	0.1	3		51.90	0.74	109.74	175.91		53.69	1.50	96.69	78.96		13.90	1.39
20	3	0.1	3		51.94	0.82	106.76	156.37		53.79	1.61	98.96	75.15		12.51	1.42
20	3	0.2	3		51.88	0.62	93.25	184.64		53.51	2.14	113.78	64.92		16.51	1.44
20	3	0.2	3		51.88	0.69	95.42	167.37		53.59	1.87	95.28	62.31		14.85	1.53
20	4	0.05	3		51.90	0.71	107.45	179.20		53.59	1.56	90.75	71.14		14.37	1.51
20	4	0.05	3		51.95	0.64	74.79	136.10		53.74	1.45	69.55	57.69		15.98	1.30
20	4	0.1	3		51.87	0.87	146.43	199.88		53.63	1.23	95.88	95.71		11.83	1.53
20	4	0.1	3		51.89	0.75	92.85	148.34		53.63	1.54	83.08	66.01		13.72	1.41
20	4	0.2	3		51.87	0.66	168.50	313.90		53.55	1.93	205.16	129.68		15.57	1.30
20	4	0.2	3		51.91	0.70	127.79	213.74		53.69	1.39	118.94	103.26		14.57	1.24
20	5	0.05	3		51.98	0.83	92.52	133.63		53.67	1.31	83.24	77.20		12.41	1.20
20	5	0.05	3		51.92	0.69	97.01	167.58		53.62	1.45	93.90	78.83		14.94	1.23
20	5	0.1	3		51.90	0.71	96.27	161.92		53.61	1.50	86.80	71.71		14.44	1.34
20	5	0.1	3		51.95	0.73	102.19	166.11		53.68	1.58	89.61	70.10		14.06	1.46
20	5	0.2	3		51.87	0.81	152.10	224.23		53.59	1.52	117.85	96.71		12.74	1.57
20	5	0.2	3		51.88	0.71	95.51	160.55		53.56	1.55	96.67	75.62		14.37	1.26
20	6	0.05	3		51.91	0.78	107.48	162.36		53.59	1.28	83.44	80.05		13.12	1.34
20	6	0.05	3		51.97	0.81	80.10	117.48		53.59	1.20	61.35	62.34		12.71	1.28
20	6	0.1	3		51.91	0.66	94.21	167.97		53.60	1.34	84.74	77.00		15.59	1.22
20	6	0.1	3		51.90	0.65	70.51	125.50		53.55	1.40	68.68	61.89		15.89	1.14
20	6	0.2	3		51.88	0.69	133.51	233.19		53.52	1.66	116.08	87.55		14.78	1.52
20	6	0.2	3		51.88	0.71	129.29	217.88		53.55	1.55	105.45	83.47		14.55	1.55

*omitted due to erroneous fitting.

Supplementary table S5. General linear model analysis of variance (ANOVA) table showing output of statistical analysis in Minitab software for pre-ageing treatment cobalt particle size. The column "Source" contains the independent variables, interactions and the error. The F-value column gives the F-value, or F-ratio, for each variable, with the general expectation that the higher this value is than 1, the less likely that the variable does not affect the dependent variable. The P-value column gives the probability that the F-ratio is larger than 1 purely due to chance. ESD is the square root of the mean square error, which should be as low as possible indicating that the error term in the model is small. R^2 and adjusted R^2 are measures of how much of the variance the model explains.

Source	Degrees of freedom	Adjusted sum of squares	Adjusted mean squares	F - value	P - value
Mg loading	7	73.09	10.44	9.32	< 0.0005
OoA	2	181.69	90.85	81.12	< 0.0005
Mg loading*OoA	14	26.80	1.91	1.71	0.062
Error	120	134.39	1.12		
Total	143				
ESD	1.06				
R^2	67.69%				
Adjusted R^2	61.50%				

Supplementary table S6. General linear model analysis of variance (ANOVA) table showing output of statistical analysis in Minitab software for pre-ageing treatment normalised cobalt peak area.

Source	Degrees of freedom	Adjusted sum of squares	Adjusted mean squares	F - value	P - value
Mg loading	7	0.42	0.06	2.71	0.012
OoA	2	0.62	0.31	13.88	< 0.0005
Mg loading*OoA	14	0.57	0.04	1.82	0.043
Error	120	2.68	0.02		
Total	143				
ESD	0.15				
R^2	37.55%				
Adjusted R^2	25.58%				

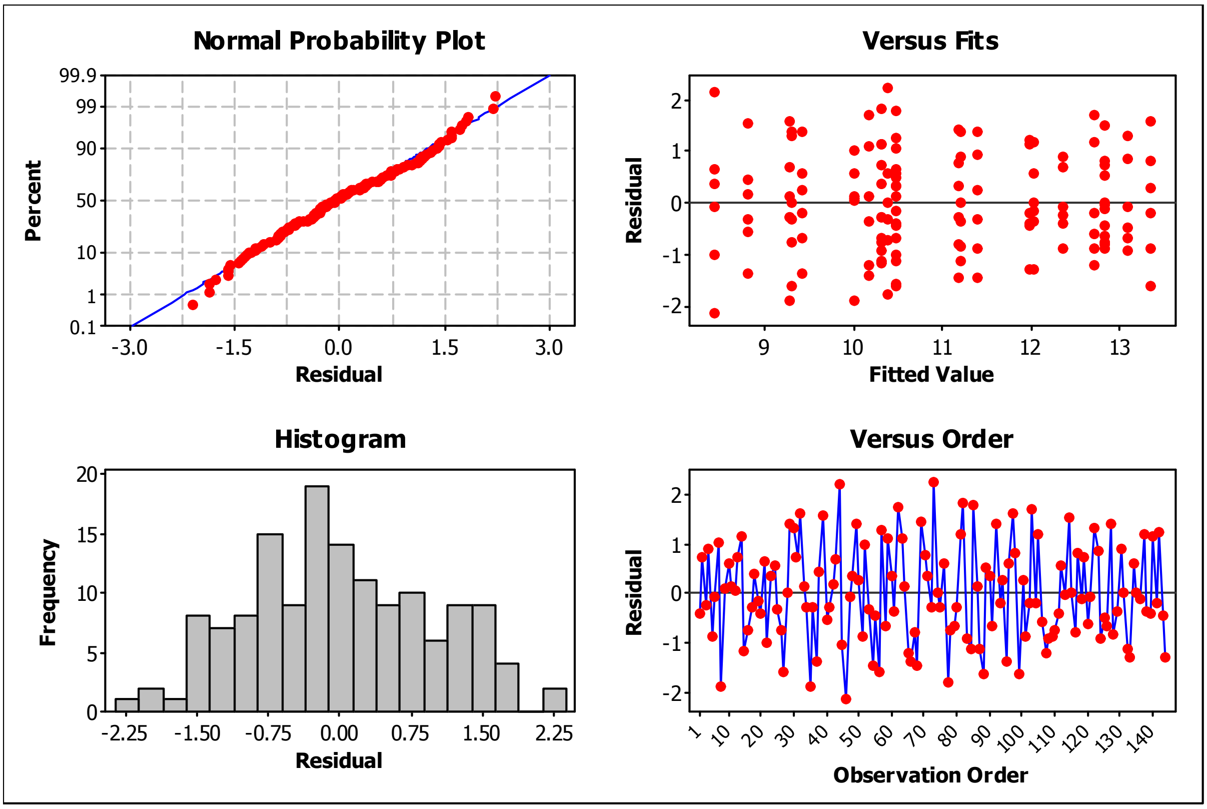

Supplementary figure S6. General linear model analysis of variance (ANOVA) residual plots showing the difference between observed and fitted values for pre-ageing treatment cobalt particle size. Various assumptions about the error term of the model are made when fitting it, and these are tested here by examining the residuals, from which the error is calculated. The top left graph tests whether the residuals are normally distributed – a departure from normality is indicated by a departure from linearity. The top right graph tests whether the variance of the residuals is constant – a violation of this would be indicated by a trend in the range of residuals. The bottom left graph tests whether the residuals are centred on zero, and the bottom right graph tests whether the errors are uncorrelated – this is indicated by there being no trend.

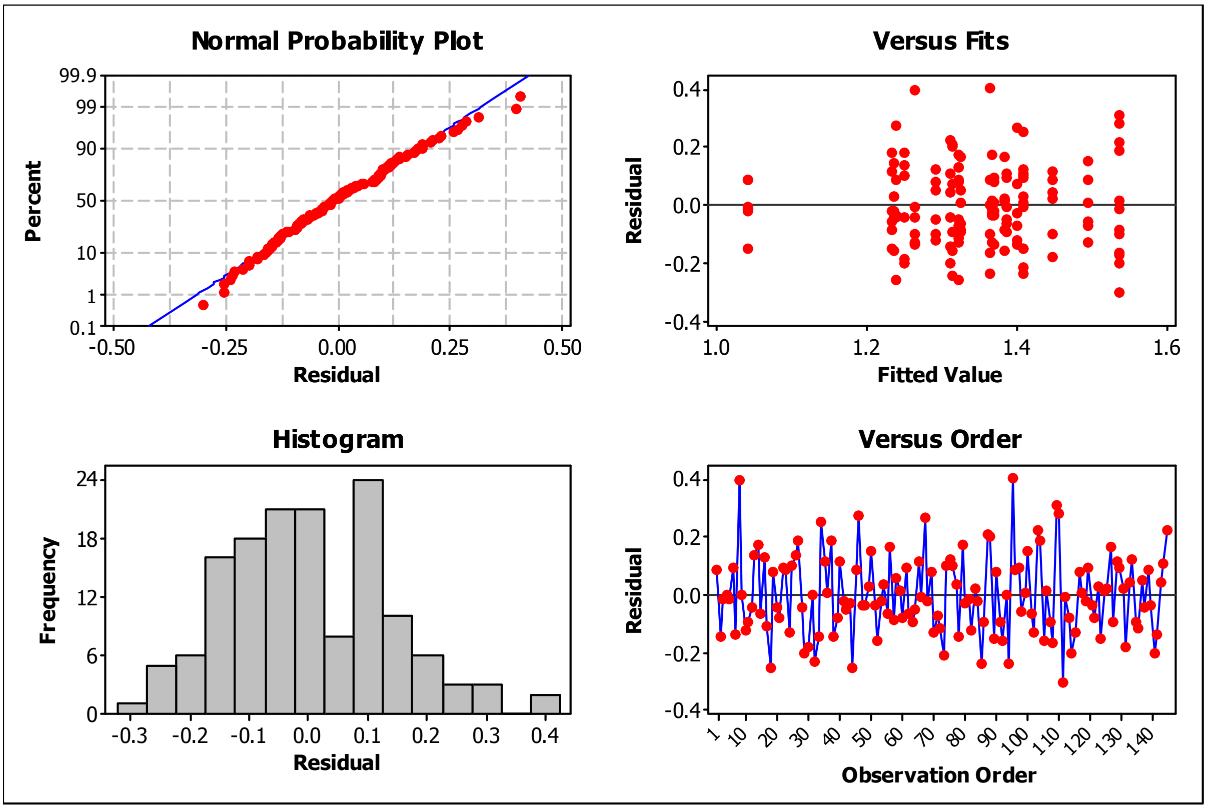

Supplementary figure S7. General linear model analysis of variance (ANOVA) residual plots showing the difference between observed and fitted values for pre-ageing treatment normalised cobalt peak area.

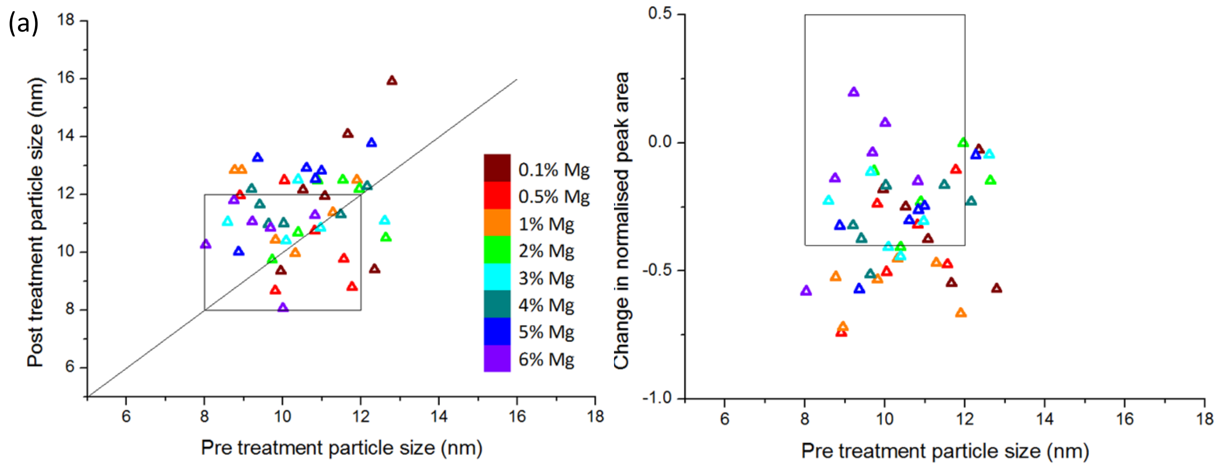

Supplementary figure S8. (a) Particle sizes before and after ageing treatment for Mg added before Co and without calcination (OoA 2). Each Mg loading has 6 data points as there are three Ru loadings and two points per unique sample. Ru loadings are not distinguished in this figure as the regression analysis showed little effect of Ru loading. Versions with Ru loadings distinguished are shown in figure S9. If points lie above the superimposed $y=x$ line, sintering has occurred. Points below the line are likely due to error (the validation work showed a standard deviation in particle size of 1.5 nm), but could also be due to metal particle reaction with support. The box shows the hit region defined by particle sizes of 8-12 nm pre-and post-ageing treatment, (b) change in normalised Co peak area against pre-treatment particle size for OoA 2 samples. The box shows the hit region defined by pre-treatment particle size of 8-12 nm and a change in normalised Co peak area of 0 to -0.4.

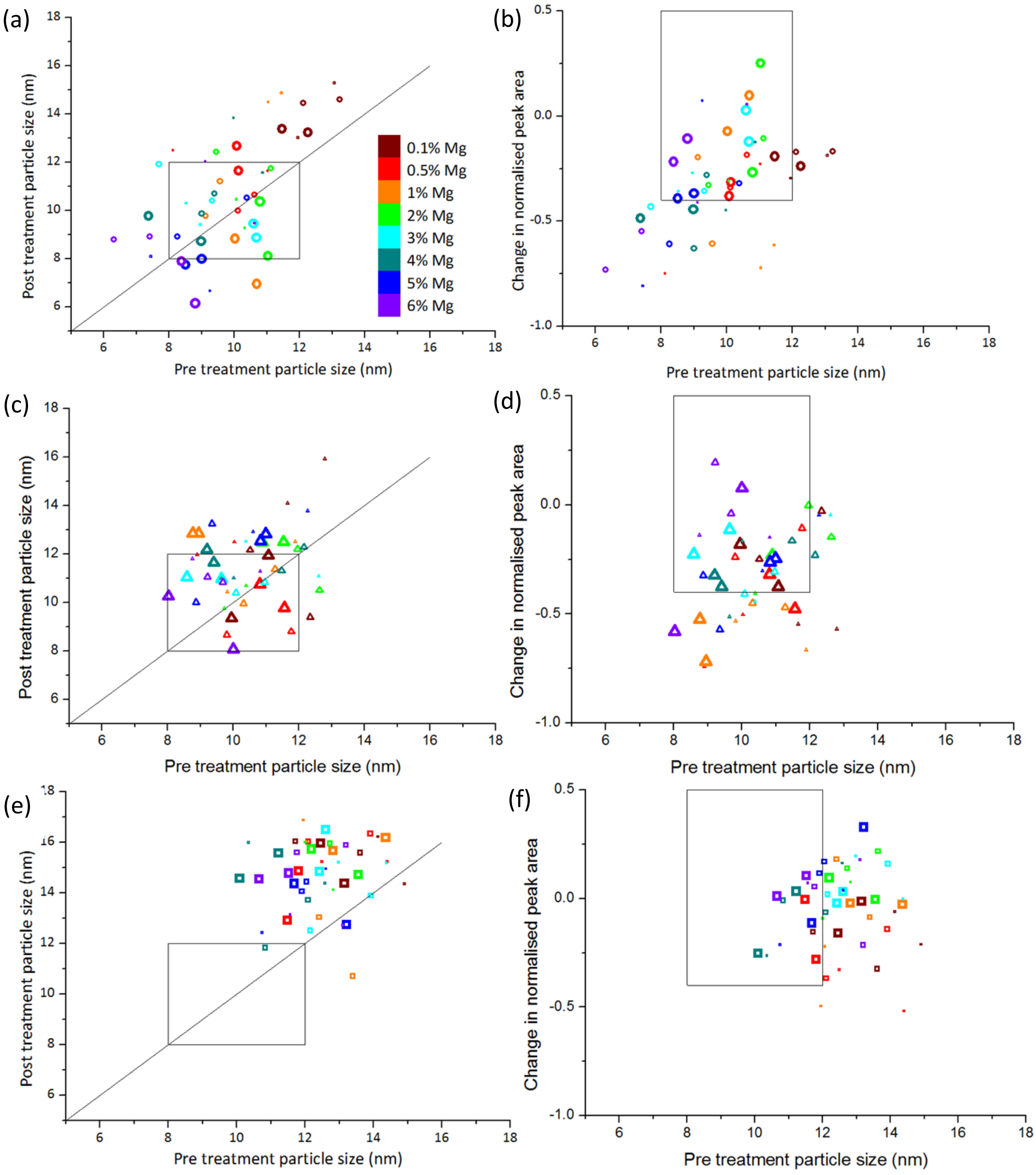

Supplementary figure S9. Equivalents of figure 3 in the main text and Supplementary figure S9, with Ru loadings distinguished by size (smallest points are 0.05%, largest are 0.2%). (a) and (b) OoA 1, (c) and (d) OoA 2 and (e) and (f) OoA 3.

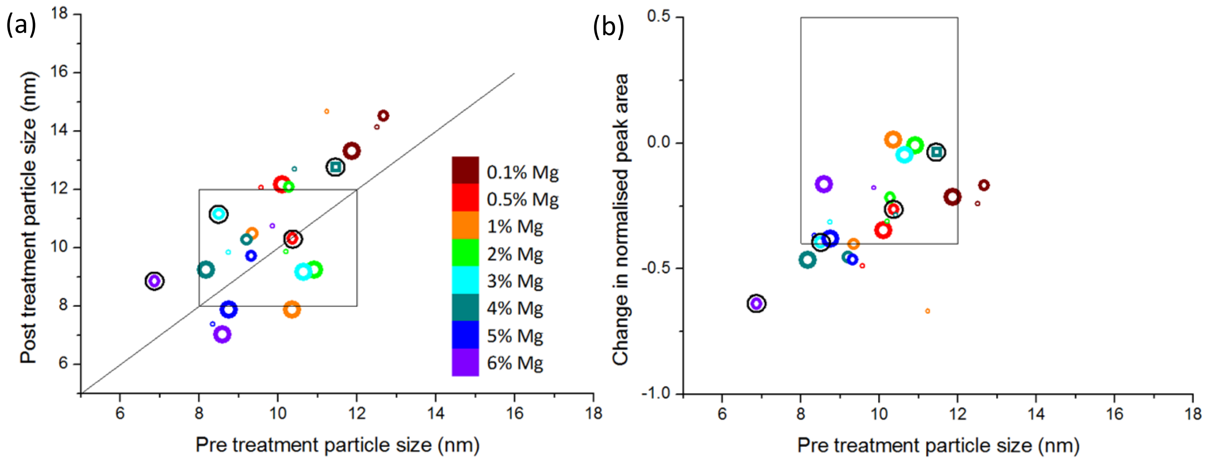

Supplementary figure S10. Equivalents to figure S9 (a) and (b) but with samples selected for scale up highlighted. The two data points for each pair have been averaged to simplify the graphs. The OoA 3 sample scaled up is shown as a square.

(a)

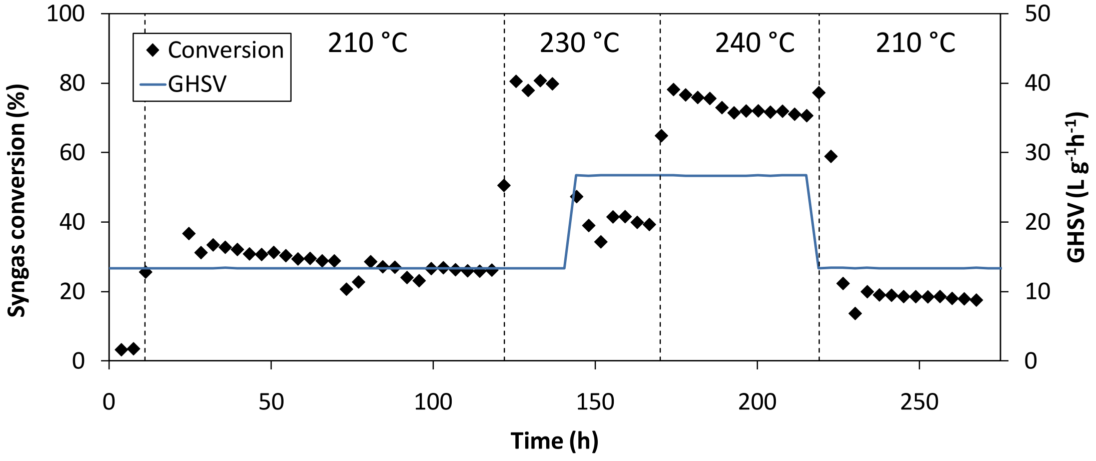

(b)

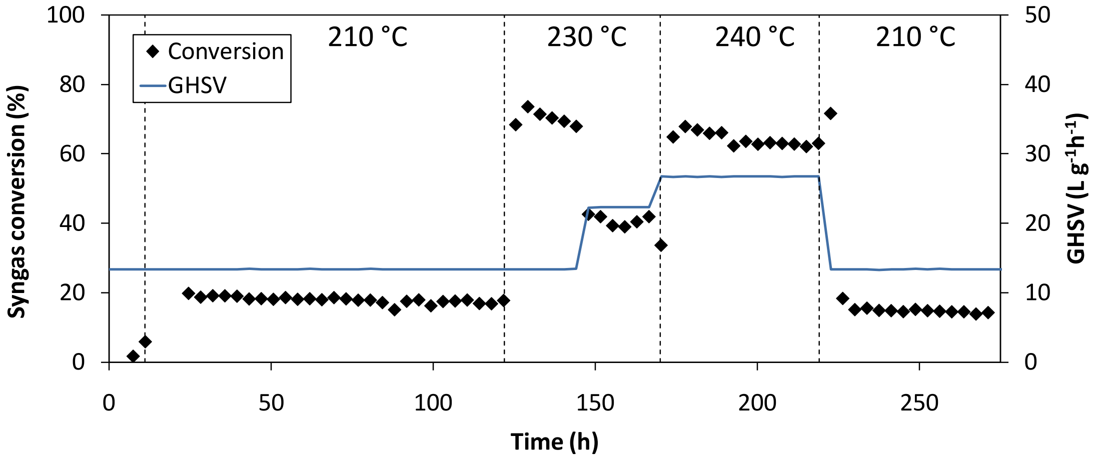

(c)

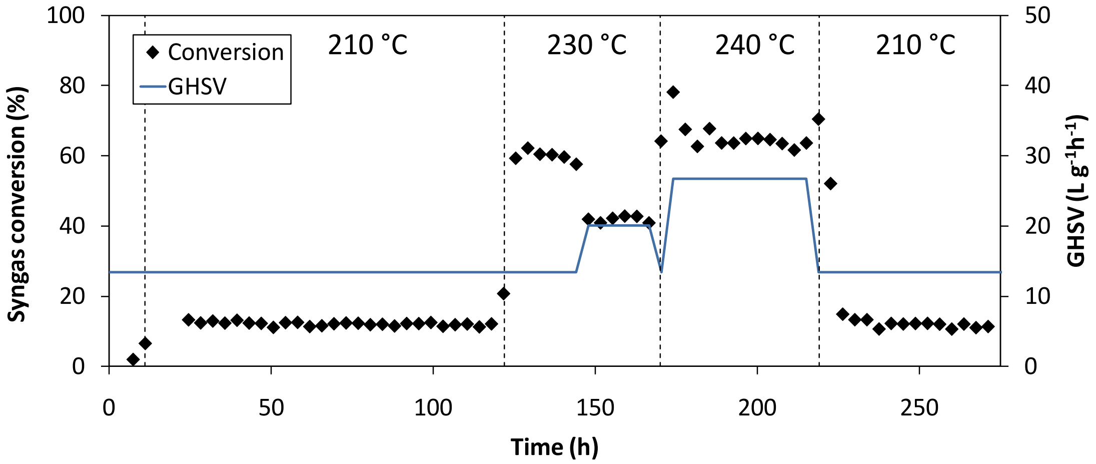

(d)

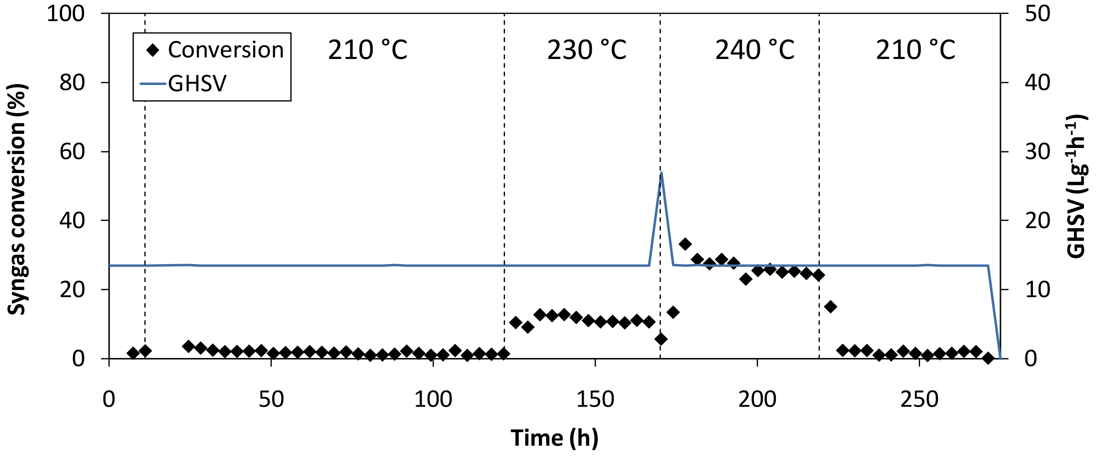

(e)

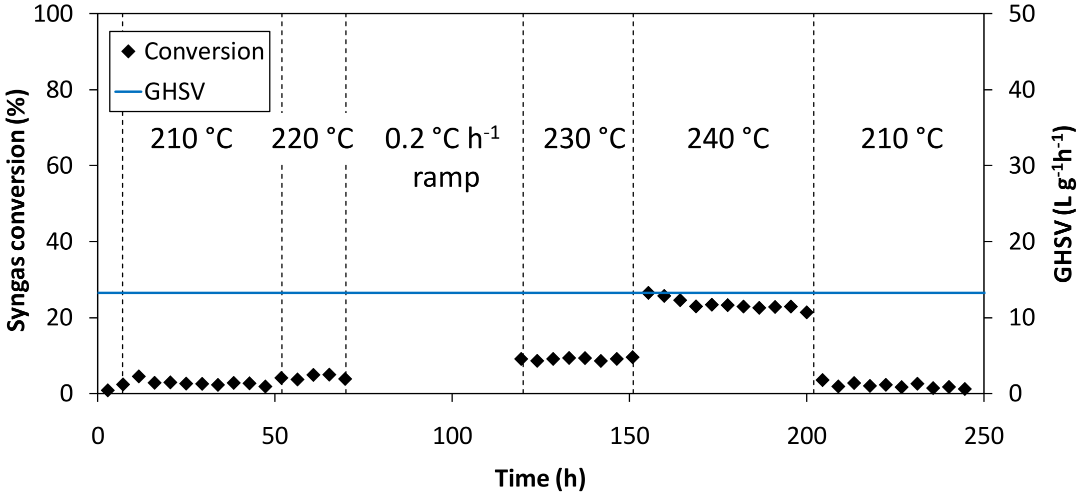

Supplementary figure S11. Catalytic testing data showing syngas conversion with time online. Samples were tested in a fixed-bed microreactor at 20 bar with $H_2/CO = 2$. Conversion was altered by control of the gas hourly space velocity (GHSV). Temperatures are shown on the plot. (a) 0% Mg (b) OoA 1, 0.5 % Mg (c) OoA 1, 3 % Mg (d) OoA 1, 6 % Mg (e) OoA 3, 4 % Mg.

(a)

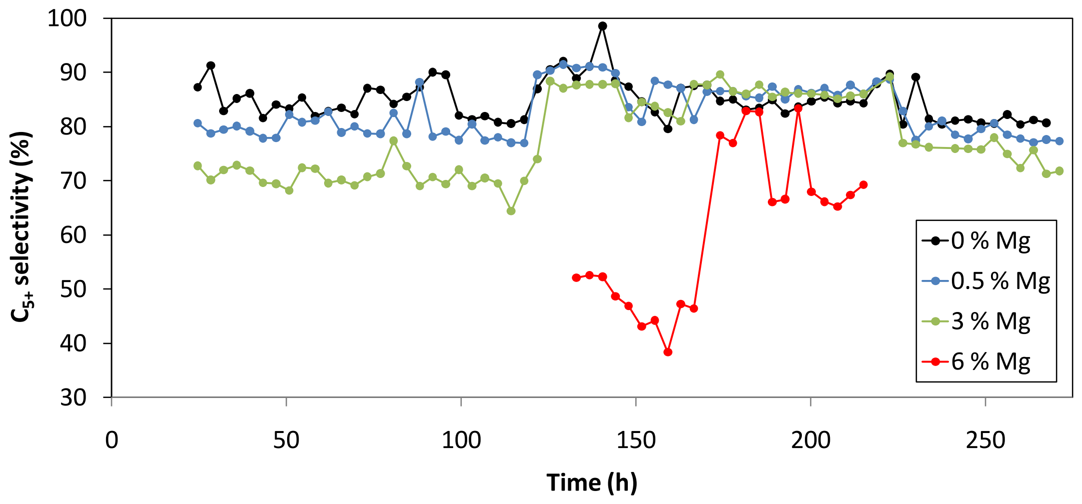

(b)

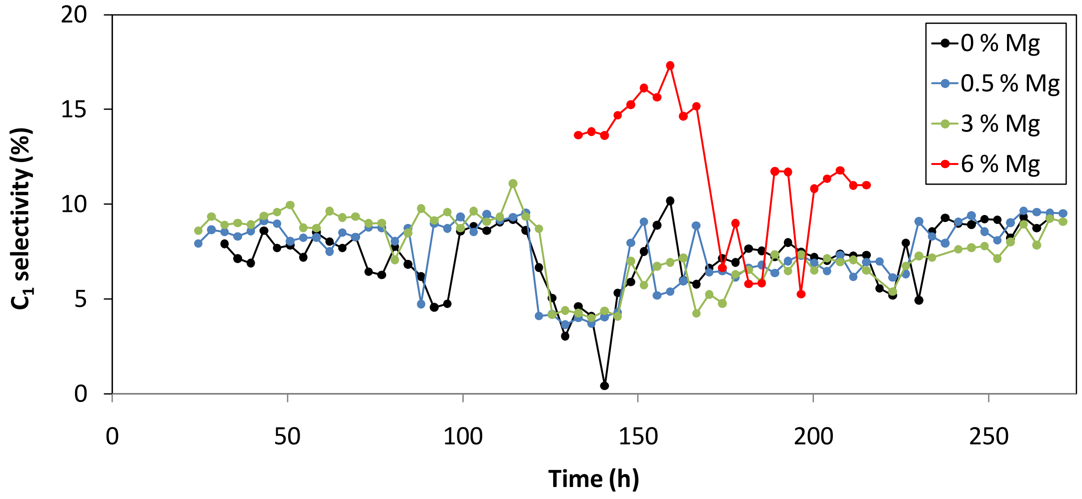

Supplementary figure S12. Catalytic testing data showing (a) Selectivity to C_{5+} products and (b) selectivity to CH_4 with time online. Samples were tested in a fixed-bed microreactor at 20 bar with $H_2/CO = 2$. Conversion was altered by control of the gas hourly space velocity (GHSV).

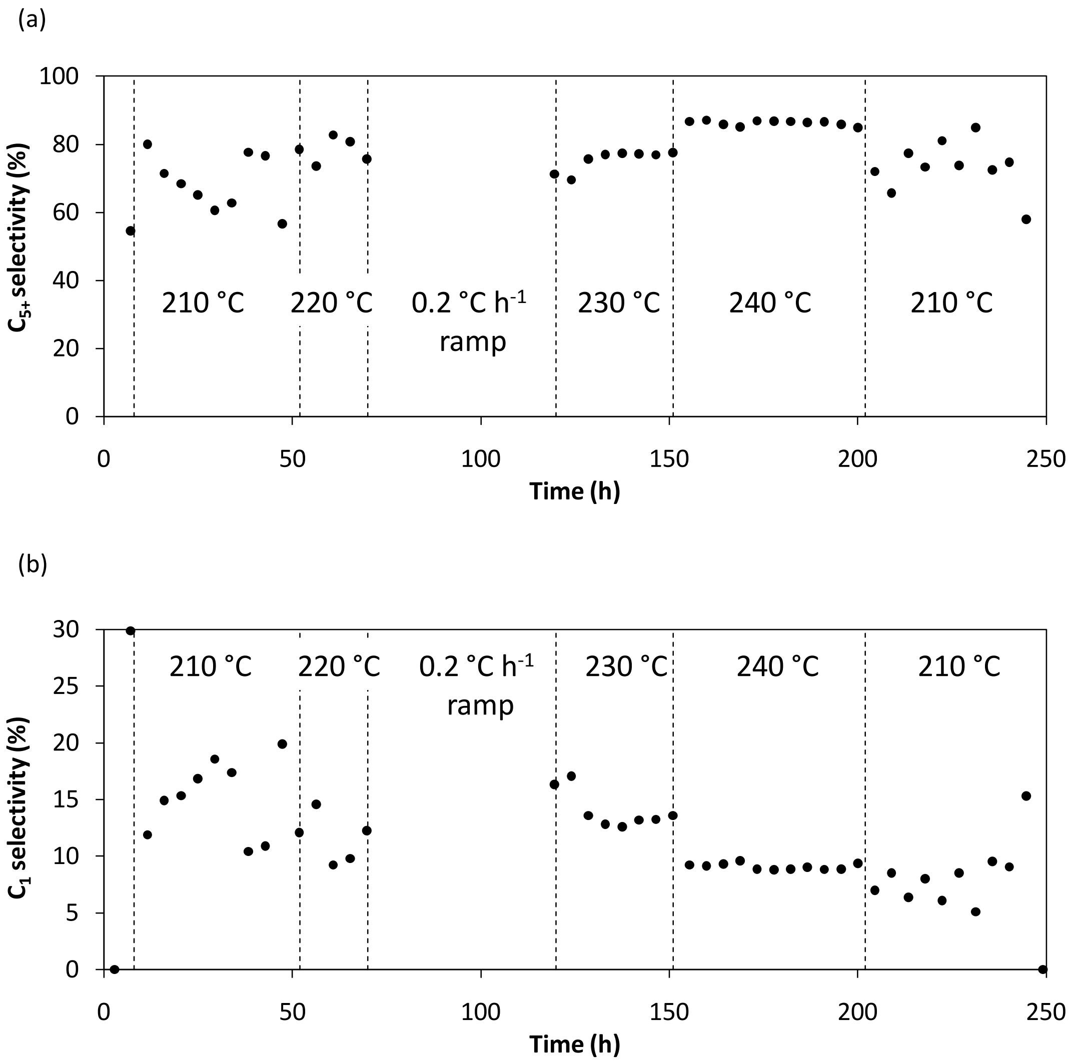

Supplementary figure S13. Catalytic testing data showing (a) Selectivity to C_{5+} products and (b) selectivity to CH_4 with time online for the scaled up 4 % Mg OoA 3 sample. Samples were tested in a fixed-bed microreactor at 20 bar with $H_2/CO = 2$.

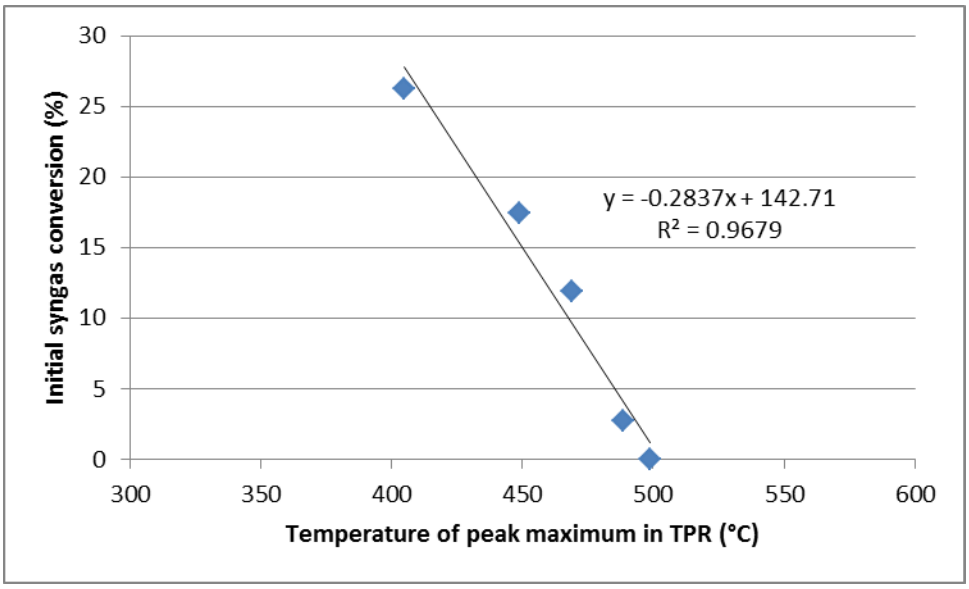

Supplementary figure S14. Plot of initial syngas conversion against temperature of peak maximum in TPR, showing the correlation between reducibility and initial activity.

References

1 Enger, B. C., Fossan, A. L., Borg, O., Rytter, E. & Holmen, A. Modified alumina as catalyst support for cobalt in the Fischer-Tropsch synthesis. *J. Catal.* 284, 9-22, doi:10.1016/j.jcat.2011.08.008 (2011).

2 StatSoft, I. *Electronic Statistics Textbook*, <http://www.statsoft.com/textbook/> (2013).

3 Abbas, H., Bale, S., Kelly, G. J. & West, J. Cobalt catalysts. WO/2010/049714A1 (2010).
